# Chemotherapy-induced microbiota exacerbates the toxicity of chemotherapy through the suppression of interleukin-10 from macrophages

**DOI:** 10.1080/19490976.2024.2319511

**Published:** 2024-02-24

**Authors:** Zhen He, Hongyu Xie, Haoyang Xu, Jinjie Wu, Wanyi Zeng, Qilang He, Christian Jobin, Sanqing Jin, Ping Lan

**Affiliations:** a Department of General Surgery (Colorectal Surgery), The Sixth Affiliated Hospital, Sun Yat-sen University, Guangzhou, Guangdong, China; b Department of Colorectal Surgery, The Sixth Affiliated Hospital, Sun Yat-sen University, Guangzhou, Guangdong, China; c Department of Anesthesia, The Sixth Affiliated Hospital, Sun Yat-sen University, Guangzhou, Guangdong, China; d School of Medicine, Shenzhen Campus of Sun Yat-sen University, Shenzhen, Guangzhou, China; e Department of Medicine, Division of Gastroenterology, University of Florida, Florida, USA; f Department of Infectious Diseases and Pathology, College of Veterinary Medicine, University of Florida, Gainesville, FL, USA; g Guangdong Provincial Key Laboratory of Colorectal and Pelvic Floor Diseases, The Sixth Affiliated Hospital, Sun Yat-sen University, Guangzhou, Guangdong, China; h Biomedical Innovation Center, The Sixth Affiliated Hospital, Sun Yat-sen University, Guangzhou, Guangdong, China; i Key Laboratory of Human Microbiome and Chronic Diseases, Sun Yat-sen University, Ministry of Education, Guangzhou, Guangdong, China; j State Key Laboratory of Oncology in South China, Guangzhou, Guangdong, China

**Keywords:** Chemotherapy-induced toxicity, microbiota, interleukin-10, macrophage

## Abstract

The gut microbiota has been shown to influence the efficacy and toxicity of chemotherapy, thereby affecting treatment outcomes. Understanding the mechanism by which microbiota affects chemotherapeutic toxicity would have a profound impact on cancer management. In this study, we report that fecal microbiota transplantation from oxaliplatin-exposed mice promotes toxicity in recipient mice. Splenic RNA sequencing and macrophage depletion experiment showed that the microbiota-induced toxicity of oxaliplatin in mice was dependent on macrophages. Furthermore, oxaliplatin-mediated toxicity was exacerbated in *Il10*^*-/-*^ mice, but not attenuated in *Rag1*^*-/-*^ mice. Adoptive transfer of macrophage into *Il10*^*-/-*^ mice confirmed the role of macrophage-derived IL-10 in the improvement of oxaliplatin-induced toxicity. Depletion of fecal *Lactobacillus* and *Bifidobacterium* was associated with the exacerbation of oxaliplatin-mediated toxicity, whereas supplementation with these probiotics alleviated chemotherapy-induced toxicity. Importantly, IL-10 administration and probiotics supplementation did not attenuate the antitumor efficacy of chemotherapy. Clinically, patients with colorectal cancer exposed to oxaliplatin exhibited downregulation of peripheral CD45^+^IL-10^+^ cells. Collectively, our findings indicate that microbiota-mediated IL-10 production influences tolerance to chemotherapy, and thus represents a potential clinical target.

## Introduction

Oxaliplatin-based chemotherapy is the first-line treatment for cancer, including colorectal cancer (CRC) and gastric cancer^1^. However, oxaliplatin often causes gastrointestinal, neural, and hematopoietic syndromes, resulting in interruption of treatment or dose reduction.^2–4^ Therefore, the amelioration of chemotherapy-induced toxicity is essential for improving cancer treatment.

Various factors have been shown to influence the toxicity of chemotherapy, including the microbiota.^5^ A recent study demonstrated that *Lachnospiraceae* and *Enterococcaceae*, together with their associated downstream metabolites (e.g., short-chain fatty acids (SCFAs) and tryptophan metabolites), could protect against radiation-induced toxicity in hematopoietic and gastrointestinal systems.^6^ In a small cohort of two patients, fecal microbiota transplantation (FMT) abrogated immune checkpoint inhibitor (ICI)-associated colitis, a phenomenon associated with reduced CD8^+^ T-cell and an increase in CD4^+^ FoxP3^+^ within the colonic mucosa.^7^ Moreover, a previous study revealed an association between the microbiome and chemotherapy-induced gastrointestinal toxicity in children with acute lymphoblastic leukemia.^8^ Severe irinotecan-induced diarrhea was also associated with alterations in intestinal microbiota composition.^9^ However, limited insight is currently available on the underlying mechanisms by which microbiota impacts chemotherapeutic-induced toxicity.

Alterations in microbiota balance have been shown to influence chemotherapy-induced inflammation and, therefore, contribute to the development of chemotherapy-associated side effects.^10^ Macrophages are important components of the innate immunity and can be regulated by various bacterial strains.^11^ During gut homeostasis, intestinal macrophages secrete various cytokines and soluble factors, including prostaglandin E2 (PGE2), bone morphogenetic protein 2 (BMP2), and WNT ligands. These molecules play crucial roles in promoting the growth of epithelial progenitor cells, regulating the function of enteric neurons, and maintaining the health of endothelial cells.^12^ A previous study demonstrated an association between macrophage polarization and capecitabine-induced hand-foot syndrome.^13^ Restoration of macrophage function, including carbon clearance, phagocytic rate, and phagocytic index, could improve the spleen and thymus index as well as enhance cell-mediated immune response, thereby ameliorating chemotherapy-induced immunotoxicity.^14^ Despite these valuable insights, microbiota-induced changes in macrophages and the mechanisms underlying chemotherapy toxicity remain unclear.

In this study, we demonstrated that microbiota-induced oxaliplatin toxicity was dependent on IL-10 secretion from macrophages. Targeted modulation of microbiota in chemotherapy-induced toxicity could improve tolerance to chemotherapy, thereby providing a precise strategy for cancer treatment.

## Results

### Alteration of gut microbiota mediates chemotherapy-induced toxicity

**Figure 1.**
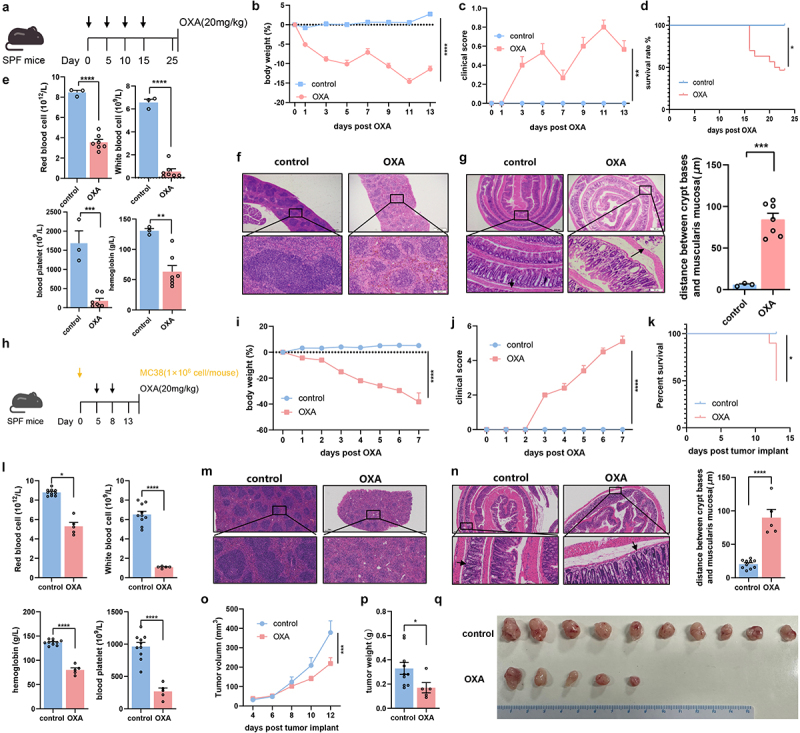
Mice exposed to high-dose oxaliplatin exhibited severe systemic side effects. (a) Oxaliplatin toxicity experimental design. SPF C57BL/6 mice were treated by oxaliplatin every five days for four times. (b-d) Changes of body weight (*p* < .0001) (b), clinical score (*p*= .0085) (c), and survival analysis after administration of oxaliplatin (*p* = .03) (d). (e) Total red blood cell count (*p* < .0001), total white blood cell count (*p* < .0001), total blood platelet count (*p* = .0001) and hemoglobin (*p* = .0033) in blood of mice with oxaliplatin intervention. (f) Representative histopathological images of spleens (Scale bars, 200 μm). (g) Representative histopathological images of colon and quantification for the gaps between crypt bases and muscularis mucosa (*p* = .0002) (Scale bars, 200 μm). Arrows indicate gaps between crypt bases and muscularis mucosa. (h) Experimental design of SPF C57BL/6 mice with injection of MC38 cells, followed by oxaliplatin intervention. (i–k) Changes of body weight (*p* < .0001) (i), clinical score (*p* < .0001) (j), and survival analysis after administration of oxaliplatin (*p* = .0122) (k). (l) Total red blood cell count (*p* = .0182), total white blood cell count (*p* < .0001), total blood platelet count (*p* < .0001) and hemoglobin (*p* < .0001) in blood of mice with oxaliplatin intervention. (m) Representative histopathological images of spleens (Scale bars, 200 μm). (n) Representative histopathological images of colon and quantification for the gaps between crypt bases and muscularis mucosa (*p*<.0001) (Scale bars, 200 μm). (o–p) Changes of tumor sizes *p* = .0003) and tumor weights (*p* = .0186) in mice treated with oxaliplatin or PBS. (h) Representative images of subcutaneous tumors from mice with treatment of oxaliplatin or PBS. Each dot indicates an individual mouse. For b-d, control: *n*=6, OXA: *n*=30. For i-k, control: *n*=10, OXA: *n*=10. The statistical significance values are denoted as: *p < .05, **p < .01, ***p < .001, ****p < .0001. Two-way ANOVA following Sidak’s multiple comparison test (b, c, i, j and o); two tailed Student t test (e, g, l, n and p); log-rank test (d and k).

To induce chemotherapy-induced toxicity, we administered a high dose of oxaliplatin (20 mg/kg) to specific pathogen-free (SPF) C57BL/6 mice every five days (Figure 1(a)). Mice exposed to high-dose oxaliplatin exhibited heighten weight loss^10^ and worse clinical scores^15^ (e.g., weight loss, hunched posture, ruffled hair coat, reluctance to move, and other performance) than those in the control group (Figures 1(b–c)). Only half of oxaliplatin-exposed mice exhibited long-term survival (20 days) (Figure 1(d)). Routine blood parameters showed that the levels of red and white blood cells, platelets, and hemoglobin significantly decreased after high-dose chemotherapy (Figure 1(e)). Moreover, oxaliplatin-exposed mice showed significantly decreased splenic white and red pulp regions (Figure 1(f)), indicating impairment of the hematopoietic system. Intestinal histological assessment showed that the gaps between the crypt bases and muscularis mucosa were significantly larger in oxaliplatin-exposed mice than in the control group (Figure 1(g)).

To further describe the chemotherapy-induced toxicity, a tumor-bearing mice model was established by subcutaneous injection of MC38 CRC cells. High dose of oxaliplatin (20 mg/kg) was subsequently injected into mice as shown in Figure 1(h). Similar to the results described above, mice exposed to the high dose of oxaliplatin exhibited an exacerbated weight loss and a worse clinical score, as well as a half of long term survival (Figures 1(i–k)). Similar change of routine blood parameters also validated the toxicity induced by chemotherapy (Figure 1(l)). Meanwhile, decreased splenic pulp regions and increased gap between the crypt bases and muscularis mucosa were found in oxaliplatin-exposed mice (Figure 1(m,n)). In addition to the toxicity associated parameters, significant alleviation of tumor growth and a corresponding reduction in tumor size and weight were observed in mice with high dose of oxaliplatin (Figures 1(o–q)). These two mice models indicate that mice exposed to high-dose oxaliplatin exhibit severe systemic side effects and toxicity to the hematopoietic and gastrointestinal systems, simulating toxicity in patients suffering from chemotherapeutics.

**Figure 2.**
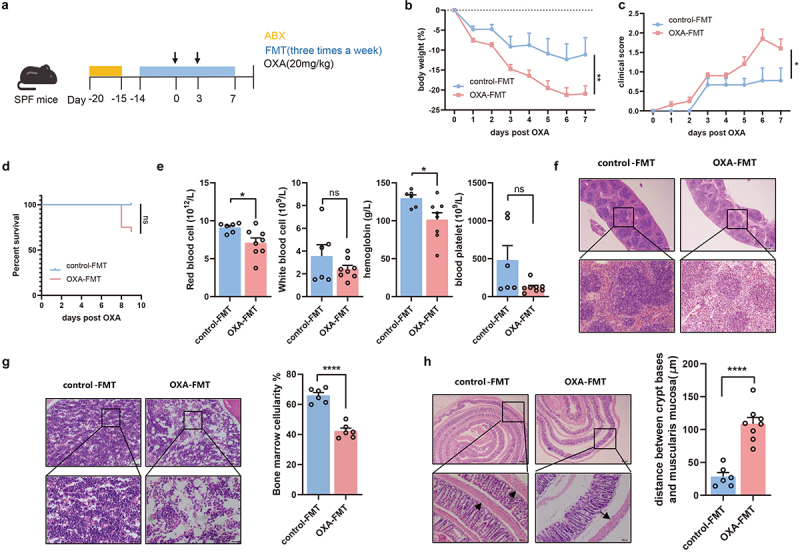
Gut microbiota altered by chemotherapy mediates the chemotherapy-induced toxicity. (a) FMT experimental design. After treated by antibiotics, mice were received FMT three times a week until end of the test. FMT recipient mice were subsequently challenged with oxaliplatin. (b–d) Changes of body weight (*p *= .0038) (b), clinical score (*p*=.0134) (c), and survival analysis (*p *= .0726) after administration of oxaliplatin (d). (e) Total red blood cell count (*p *= .0252), total white blood cell count (*p *= .2546), hemoglobin in blood (*p *= .0291) and total blood platelet count (*p *= .0523) of mice with FMT. (f) Histopathological images of spleens (Scale bars, 200 μm). (g) Femurs from mice with FMT were stained with H&E and quantification for bone marrow cellularity (Scale bars, 100 μm). (h) Histopathological images of colon and quantification for the gaps between crypt bases and muscularis mucosa (*p *< .0001) (Scale bars, 200 μm). Arrows indicate gaps between crypt bases and muscularis mucosa. Each dot indicates an individual mouse. For b-d, control-FMT: *n*=9, OXA-FMT: *n*=20. The statistical significance values are denoted as: *p < .05, ** *p *< .001, **** *p *< .0001. Two-way ANOVA following Sidak’s multiple comparison test (b, c); two tailed student t test (e, g, and h); log-rank test (d).

To explore whether gut microbiota has a causal effect on chemotherapy-induced toxicity, we collected feces from mice without subcutaneous tumors treated with high doses of oxaliplatin and performed fecal microbiota transplantation (FMT) in healthy C57BL/6 recipient mice. We also demonstrated that there was no oxaliplatin detected in feces from mice given multiple intraperitoneal injections of oxaliplatin by liquid chromatography-mass spectrometry (LC-MS) (Figure S1(a)). All recipient mice (OXA-FMT and control-FMT) were challenged with oxaliplatin (Figure 2(a)). Interestingly, OXA-FMT mice exhibited heightened weight loss, worse clinical scores, shorter survival durations, and worse routine blood parameters than the control-FMT group (Figures 2(b–e)). Histological analysis further showed a significant decrease in bone marrow cellularity and splenic loss of white and red pulp regions, as well as larger gaps between the crypt bases and muscularis mucosa in the OXA-FMT group than in the control-FMT group (Figures 2(f–h)). These findings demonstrate that the microbiota from oxaliplatin-exposed mice exacerbates chemotherapy-induced toxicity in recipient mice.

### Microbiota-mediated toxicity of chemotherapy is macrophage-dependent

**Figure 3.**
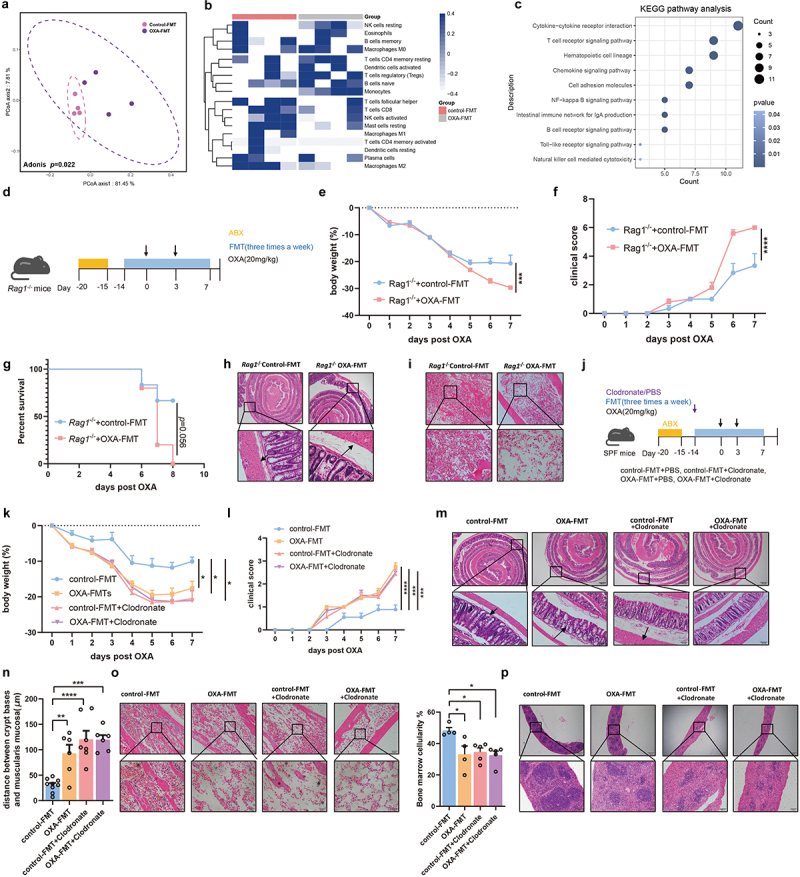
Microbiota-mediated toxicity of chemotherapy is macrophage-dependent. (a) Splenic transcriptome from recipient mice with FMT, revealed by PCoA (adonis *p *= .022). (b) Distribution of splenic immune cells was revealed by transcriptome. (c) Pathway analysis of the immune-associated differential expression genes. (d) After treatment of antibiotics cocktail, *Rag1*^*-/-*^ mice with FMT were injected with oxaliplatin. (e–g) Changes of body weight (*p*=.0004) (e), clinical score (*p *< .0001) (f), and survival analysis (g) after administration of oxaliplatin. (h) Histopathological images of colon. Arrows indicate gaps between crypt bases and muscularis mucosa (Scale bars, 200 μm). (i) Femurs from *Rag1*^*-/-*^ mice were stained with H&E (Scale bars, 100 μm). (j) Experimental design of macrophage depletion. Mice with antibiotics cocktail intervention were treated with clodronate liposomal or PBS liposomal, followed by FMT treatment and oxaliplatin treatment. (k–l) Changes of body weight (control-FMT vs. OXA-FMT: *p *= .0298, control-FMT vs. control-FMT+Clodronate: *p *= .02, control-FMT vs. OXA-FMT+Clodronate: *p *= .0343) (k) and clinical score (control-FMT vs. OXA-FMT: *p *< .0001, control-FMT vs. control-FMT+Clodronate: *p *= .0005, control-FMT vs. OXA-FMT+Clodronate: *p *= .0009) (l) after administration of oxaliplatin. (m,n) Histopathological images of colon and quantification for the gaps between crypt bases and muscularis mucosa (Scale bars, 200 μm). Arrows indicate gaps between crypt bases and muscularis mucosa (control-FMT vs. OXA-FMT: *p*=.0057, control-FMT vs. control-FMT+Clodronate: *p *< .0001, control-FMT vs. OXA-FMT+Clodronate: *p *= .0001). (o) Femurs from mice with FMT were stained with H&E and quantified for bone marrow cellularity (control-FMT vs. OXA-FMT: *p *= .0338, control-FMT vs. control-FMT+Clodronate: *p *= .0433, control-FMT vs. OXA-FMT+Clodronate: *p *= .0193) (Scale bars, 100 μm). (p) Histopathological images of spleens (Scale bars, 200 μm). Each dot indicates an individual mouse. For a-d, control-FMT: *n*=4, OXA-FMT: *n*=4. For f-g, Rag1^−/−^+control-FMT: *n*=6, Rag1^−/−^+OXA-FMT: *n*=5. For k-l, control-FMT: *n*=9, OXA-FMT: *n*=8, control-FMT+Clodronate: *n*=7, OXA-FMT+Clodronate: *n*=6. The statistical significance values are denoted as: * *p *< .05, ** *p *< .01, *** *p *< .001, **** *p *< .0001. Two-way ANOVA following Sidak’s multiple comparisons test (e and f); log-rank test (g); one-way ANOVA following Tukey’s multiple comparison test (n and o); two-way ANOVA following Tukey’s multiple comparison test (k and l).

To clarify the mechanisms by which the microbiota mediates chemotherapy-induced toxicity, we performed transcriptional analysis of splenic cells obtained from OXA-FMT mice and control group. The splenic transcriptome of the OXA-FMT group was significantly different from that of the control-FMT group (Figure 3(a)). We then evaluated the relative abundance of different immune cells using the CIBERSORT algorithm and found that the fraction of monocytes and macrophages changed significantly (Figure 3(b)). An increased proportion of monocytes and decreased proportion of M2 macrophages were observed in the OXA-FMT group (Figure 3(b)). Alteration of several differential genes associated with monocytes and M2 macrophages was also observed after FMT intervention (Figure S1(b)). Furthermore, immunohistochemistry (IHC) indicated that the proportion of macrophages decreased, rather than the proportion of CD4^+^ T cells and Treg cells (Figures S1(c–h)). We further evaluated the differential genes referred in CIBERSORT algorithm. In addition to the involvement of different immune cell signaling pathways, we found that the differential genes were also clustered in the Toll-like receptor signaling pathway and NF-κB signaling pathway (Figure 3(c)).

To subsequent confirm the role of immune cell in chemotherapy-induced toxicity, we next applied FMT experiments with recombination activating gene 1 (*Rag-1*)-deficient (*Rag1*^*-/-*^) mice lacking mature B and T lymphocytes (Figure 3(d)). *Rag1*^*-/-*^ mice gavaged with feces from oxaliplatin-treated donors exhibited greater body weight loss, higher clinical scores, and shorter survival durations (Figures 3(e–i)), implicating the other potential immune response such as innate immunity were involved in microbiota-mediated chemotherapy toxicity. While immunologic memory is a key feature of adaptive immunity, more recently the term “trained innate immunity” has been used to describe innate immune cells, primarily macrophages that exhibit enhanced responsiveness upon reinfection.^16^ To investigate the role of macrophages in chemotherapy-induced toxicity, recipient mice were intraperitoneally injected with clodronate to eliminate macrophages, followed by FMT, as described above (Figure 3(j)). Flow cytometric analysis confirmed that macrophages were depleted in splenic cells of mice treated with clodronate (Figure S1(i)). Mice with undepleted macrophages exhibited heightened chemotherapeutic-induced toxicity in the OXA-FMT group compared to the control-FMT group. Interestingly, similar weight loss, clinical score, and pathologic features were observed between OXA-FMT and control-FMT in macrophage-depleted recipient mice, which were both significantly lower than those in mice with intact macrophages (Figures 3(k–p)). Collectively, these findings demonstrate that microbiota-mediated chemotherapy-induced toxicity is dependent on macrophage function.

### Suppression of IL-10 is responsible for chemotherapy-induced toxicity

**Figure 4.**
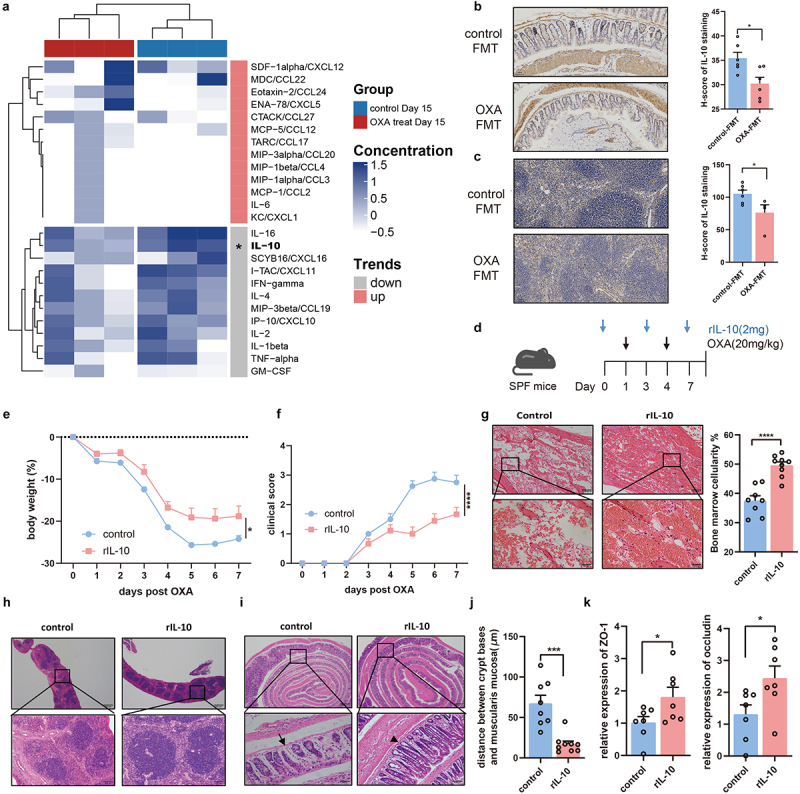
Suppression of IL-10 is responsible for chemotherapy-induced toxicity. (a) Cytokine/chemokine profile of the serum from mice with FMT. An asterisk (*) indicated the significant change of cytokine. (b) In colon tissue, the immunohistochemical staining of IL-10 (*p *= .0145) was analyzed from the perspective of histological grades (H score) (Scale bars, 200 μm). (c) In spleen tissue, the immunohistochemical staining of IL-10 (*p *= .0426) was analyzed from the perspective of histological grades (H score) (Scale bars, 200 μm). (d) Experimental design of supplement rIL-10 or PBS for SPF C57BL/6 mice, followed by oxaliplatin intervention. (e,f) Changes of body weight (*p *= .0347) (e) and clinical score (*p *< .0001) (f) after administration of oxaliplatin. (g) Femurs from mice with rIL-10 or PBS supplement were stained with H&E and quantified for bone marrow cellularity (*p *< .0001) (Scale bars, 100 μm). (h) Histopathological images of spleens (Scale bars, 200 μm). (i–j) Histopathological images of colon (Scale bars, 200 μm) and quantification for the gaps between crypt bases and muscularis mucosa (*p *= .0002). Arrows indicate gaps between crypt bases and muscularis mucosa. (k) Relative mRNA levels of ZO-1 (*p *= .0478) and occluding (*p *= .0370) in the colon from mice with rIL-10 or PBS supplement. Each dot indicates an individual mouse. For a, control-FMT: *n*=2, OXA-FMT: *n*=2. For e-f, control: *n*=8, rIL-10: *n*=9. The statistical significance values are denoted as: *p < .05,   ***p < .001, **** *p *< .0001. Two-way ANOVA following Sidak’s multiple comparison test (e and f); two tailed student t test (b, c, g, j, and k).

To identify the most prominent immune response induced by the gut microbiota in chemotherapy-induced toxicity, we measured the expression of 31 serum cytokines in the OXA-FMT and control-FMT mice (Figure 4(a)). Mice colonized with oxaliplatin-treated microbiota exhibited different serum cytokine levels compared with control-FMT mice. Specifically, significant downregulation of IL-10 was observed in mice colonized with oxaliplatin-treated microbiota (Figure 4(a)). Moreover, IHC experiments confirmed that the expression of IL-10 in the colon and spleen of OXA-FMT group mice was significantly decreased compared to that in the control-FMT group (Figures 4(b–c)). IL-10 is an important cytokine that suppresses the inflammatory response. To explore the role of IL-10 in chemotherapy-induced toxicity, we intraperitoneally injected oxaliplatin into *Il10*^*-/-*^ and wild-type (WT) mice (Figure S2(a)). Interestingly, *Il10*^*-/-*^ mice exhibited worse weight loss and clinical scores, as well as the exacerbation of histological features (Figures S2(b–f)). To confirm the role of IL-10, we intraperitoneally injected recombinant IL-10 (rIL-10) into C57BL/6 mice (Figure 4(d)). Mice administered rIL-10 exhibited significantly lower weight loss and improved clinical scores upon oxaliplatin exposure (Figures 4(e–f)). Additionally, administration of rIL-10 also rescued the exacerbation of splenic white and red pulp regions as well as the gaps between the crypt bases and muscularis mucosa, indicating an improvement in hematopoietic and gastrointestinal toxicity (Figures 4(g–j)). Moreover, rIL-10 treatment increased the mRNA levels of epithelial tight junctions, such as ZO-1 and occludin in the colon, suggesting increased barrier function (Figure 4(k)). These data demonstrate that the microbiota-mediated downregulation of IL-10 expression is responsible for the exacerbation of chemotherapy-induced toxicity.

### Downregulation of IL-10 from macrophage mediates the chemotherapy-induced toxicity

**Figure 5.**
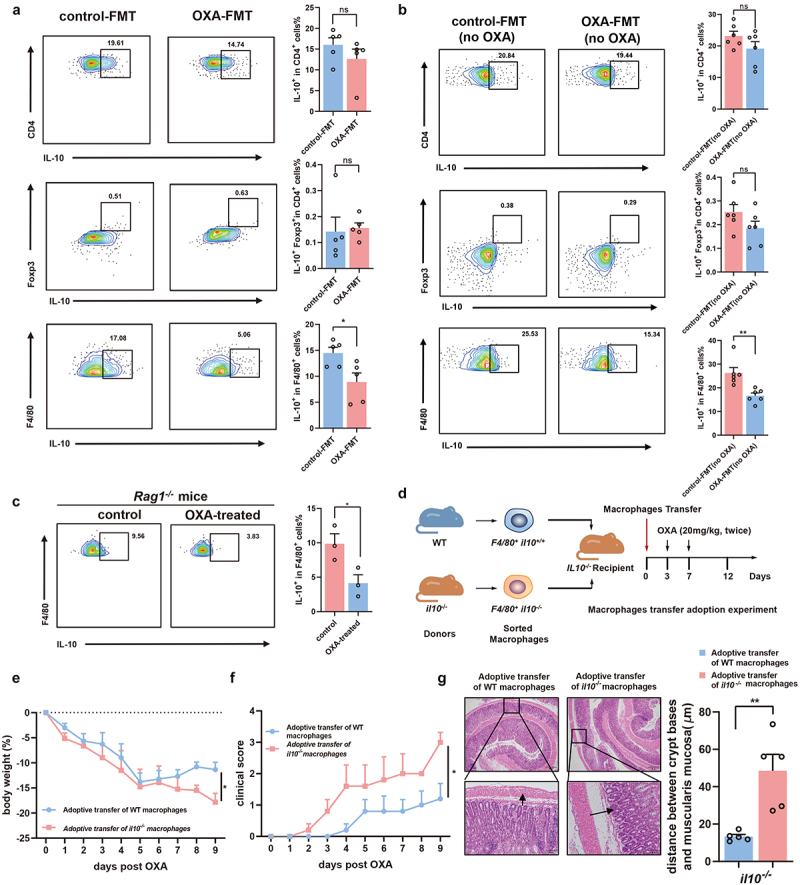
Chemotherapy toxicity-associated IL-10 secretion in macrophages is through TLR4. (a) IL-10 secretion from CD4^+^ cells (*p *= .2838), Treg cells (*p *= .8194), and macrophages (*p *= .0265) in control-FMT mice and OXA-FMT mice were analyzed by flow cytometry. (b) IL-10 secretion from CD4^+^ cells (*p *= .1639), Treg cells (*p *= .1432), and macrophages (*p *= .0041) in control-FMT (no OXA) mice and OXA-FMT (no OXA) mice were analyzed by flow cytometry. (c) Splenocytes from *Rag1*^*-/-*^ mice were stimulated by feces supernatant from control or oxaliplatin-treated mice for 24 hours. Expression of IL-10 in F4/80^+^ macrophages were analyzed by flow cytometry (*p *= .0391). (d) Adoptive transfer of macrophage mice model. F4/80^+^ macrophages were isolated from *Il10*^*-/-*^ and WT mice respectively. Isolated macrophages were transferred into *Il10*^*-/-*^ mice followed by twice of oxaliplatin treatment. (e,f) Changes of body weight (*p *= .0252) (e) and clinical score (*p *= .0188) (f) after administration of oxaliplatin. (g) Histopathological images of colon and quantification for the gaps between crypt bases and muscularis mucosa (*p *= .0042) (Scale bars, 200 μm). Arrows indicate gaps between crypt bases and muscularis mucosa. Each dot indicates an individual mouse. For d, control recipient: *n*=5, *Il10*^*-/-*^ recipient: *n*=5. The statistical significance values are denoted as: *p < .05, **p < .01   . Two tailed Student t test (a, b, c and g). Two-way ANOVA following Sidak’s multiple comparison test (e and f).

We next determined the source of IL-10 secretion by flow cytometry. Flow cytometry analysis of splenocytes showed that F4/80^+^ IL-10^+^ macrophages were markedly suppressed in recipient mice in the OXA-FMT group compared with those in the control-FMT group (Figure 5(a)). Interestingly, both CD4^+^ IL-10^+^ T cells and CD4^+^Foxp3^+^ IL-10^+^ regulatory T cells in splenocytes were similar between this two groups (Figure 5(a)). Additionally, we assessed whether FMT treatment in the absence of oxaliplatin exposure led to similar changes (Figure S3(a)). Although there was no difference in body weight between the control-FMT (FMT only, no oxaliplatin treatment) group and OXA-FMT (FMT only, no oxaliplatin treatment) group (Figure S3(b)), flow cytometry analysis of splenocytes showed that the changes of F4/80^+^ IL-10^+^ macrophages, CD4^+^ IL-10^+^ T cells, and CD4^+^Foxp3^+^ IL-10^+^ regulatory T cells in splenocyte were consistent with the FMT-OXA-exposure experiment results (Figure 5(b)).

To assess the impact of microbiota on macrophage-derived IL-10 secretion, we isolated splenocytes from *Rag1*^*-/-*^ mice and co-cultured them for 24 h with fecal supernatants obtained from control or oxaliplatin-treated mice. Flow cytometry analysis revealed that the number of F4/80^+^ IL-10^+^ cells significantly decreased after treatment with the fecal supernatant from oxaliplatin-treated mice (Figure 5(c)). To further confirm the role of macrophage-derived IL-10 in chemotherapy-induced toxicity, we further established mice model with adoptive transfer of macrophage. F4/80^+^ macrophages were isolated from *Il10*^*-/-*^ and WT mice respectively. These isolated macrophages were transferred into *Il10*^*-/-*^ mice followed by twice of oxaliplatin treatment (Figure 5(d)). Compared with the *Il10*^*-/-*^ mice with F4/80^+^IL-10^−/−^ macrophages, mice adopted with F4/80^+^ IL-10^+/+^ macrophages exhibited a significantly improved weight loss and clinical scores after high dose of oxaliplatin treatment (Figure 5(e,f)). Similar to the phenotype changes, histological analysis also revealed the improved toxicity in the gastrointestinal systems in *Il10*^*-/-*^ mice with F4/80^+^ IL-10^+/+^ macrophages adoption (Figures 5(g)).

To explore the downstream changes in macrophage, we next analyzed the splenic transcriptome. Differential genes associated with microbial antigen presentation, such as Toll-like receptor 4 (*TLR4*), *TLR9*, *TLR12*, *CD40*, *CCL4*, and *CARD11*, were significantly downregulated in the OXA-FMT group compared to control-FMT group (Figure S3(c)). Downstream signaling molecules associated with the NF-κB signaling pathway, such as *IKBKB*, *IKBKG*, *TNFSF14*, *TRAF3*, and *TRAF5*, were consistently downregulated (Figure S3(c)). These alteration was validated by qPCR analysis (Figure S3(d)). Specifically, TLR4 signaling plays an essential role in bacteria-induced innate immune responses. Similar to the splenic transcriptome, mRNA expression of *TLR4*, *Myd88*, *NFKB1A* and *IL-10* was significantly downregulated in RAW264.7 murine macrophage cell line and bone marrow-derived macrophages (BMDMs) stimulated with fecal supernatant from oxaliplatin-treated mice (Figures S3(e-f)). To further confirm the role of TLR4 in IL-10 secretion, we isolated splenocytes from *tlr4*^*Lps-del*^ mice in which harbored dysfunction of TLR4. These isolated splenocytes were exposed to fecal supernatant from oxaliplatin-treated mice. The number of F4/80^+^ IL-10^+^ cells was significantly decreased in *tlr4*^*Lps-del*^ mice compared to that in WT mice (Figure S3(g,h)). These data suggest that microbiota-mediated chemotherapy-induced toxicity is associated with the suppression of TLR4-IL-10 signaling pathway in macrophage.

### Chemotherapy-induced toxicity is associated with depletion of bacteria with probiotic properties

**Figure 6.**
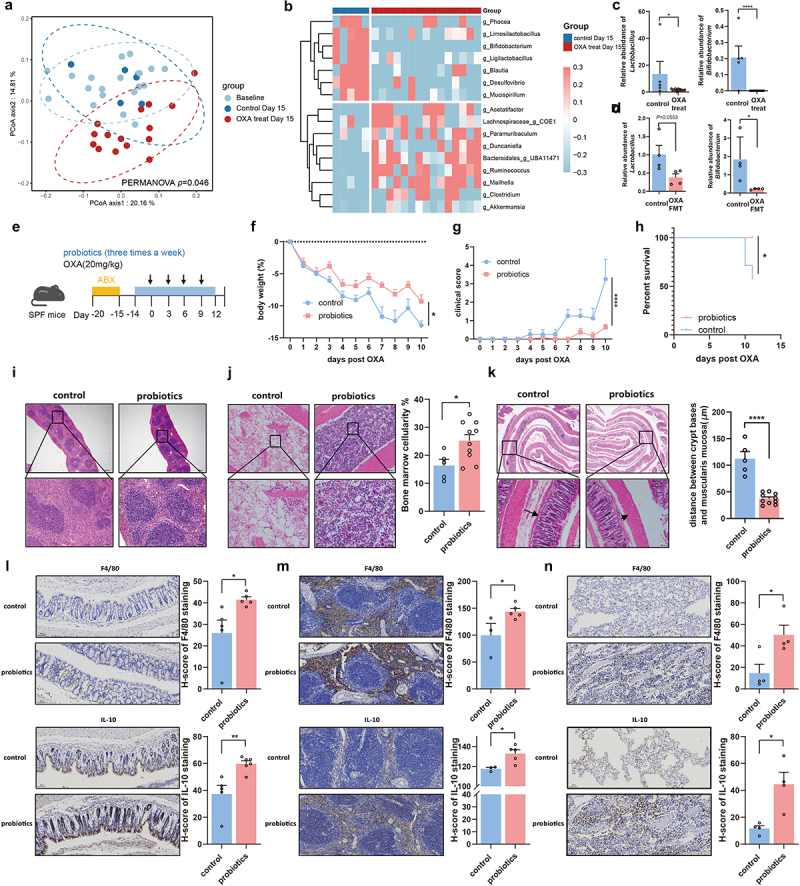
Restoration of microbiota-depleted probiotics alleviates chemotherapy-induced toxicity. (a) PCoA of the gut microbiota based on the permutational multivariate analysis of variance (PERMANOVA *p *= .046). (b) Differential analysis indicated the enrichment of bacteria in mice from oxaliplatin-treated group and control group. (c) Relative abundance of *Lactobacillus* (*p *= .0322) and *Bifidobacterium*(*p *< .0001) in mice from the control and oxaliplatin-treated group. (d) Relative abundance of *Lactobacillus* (*p *= .0553) and *Bifidobacterium* (*p *= .0386) in the control-FMT and OXA-FMT mice by qPCR detection. (e) Experimental design of SPF C57BL/6 mice with probiotics supplement, followed by oxaliplatin treatment. (f-h) Changes of body weight (*p *= .0220) (f), clinical score (*p *< .0001) (g), and survival analysis (*p *= .0189) (h) after administration of oxaliplatin. (i) Histopathological images of spleens (Scale bars, 200 μm). (j) Femurs from mice with probiotics treatment were stained with H&E (Scale bars, 100 μm) and quantification for bone marrow cellularity (*p *= .0233). (k) Histopathological images of colon (Scale bars, 200 μm) and quantification for the gaps between crypt bases and muscularis mucosa (*p *< .0001). Arrows indicate gaps between crypt bases and muscularis mucosa. (l) In colon tissue, the immunohistochemical staining of F4/80 (*p *= .0366), and IL-10 (*p *= .0056) was analyzed from the perspective of histological grades (H score) (Scale bars, 200 μm). (m) In spleen tissue, the immunohistochemical staining of F4/80 (*p *= .0471), and IL-10 (*p *= .0245) was analyzed from the perspective of histological grades (H score) (Scale bars, 200 μm). (n) In femur tissue, the immunohistochemical staining of F4/80 (*p *= .0272), and IL-10 (*p *= .0117) was analyzed from the perspective of histological grades (H score) (Scale bars, 100 μm). Each dot indicates an individual mouse. For a and b, baseline: *n*=20, control Day15: *n*=5, OXA-treated Day15: *n*=15. For f-h, control: *n*=7, probiotics: *n*=11. The statistical significance values are denoted as: *p < .05, **p < .01,  ****p < .0001. Two-way ANOVA following Sidak’s multiple comparison test (f and g); two tailed student t test test (c, d, j, k, and l-n); log-rank test (h).

Our results suggested that oxaliplatin causes cellular toxicity by impairing microbiota-induced IL-10 expression in macrophages. To gain more insight into microbiota alterations, we determined the bacterial composition in mice treated with oxaliplatin or PBS using 16S rRNA sequencing. Compared with the microbial feature in baseline and control group, our taxonomic analysis of the microbiome using principal coordinate analysis (PCoA) showed a significant clustering and separation in mice treated with oxaliplatin (Figure 6(a)). We subsequently detected marked differences in the bacterial community abundance after oxaliplatin treatment. Notably, several genera with probiotic properties, such as *Lactobacillus* (*Limosilactobacillus and Ligilactobacillus*), *Bifidobacterium* and *Blautia* were significantly depleted in oxaliplatin-treated mice, while some genera including *Ruminococcus*, *Paramuribaculum* and *Clostridium* were enriched in oxaliplatin-treated mice (Figure 6(b)). We subsequently applied qPCR analysis to validate the changes of probiotics. Our results confirmed that the relative abundance of *Lactobacillus* and *Bifidobacterium* were lower in the feces of oxaliplatin-treated mice and recipients that received FMT from oxaliplatin-treated donors (Figures 6(c,d)). These data demonstrate that the toxicity of chemotherapy is associated with the depletion of bacteria with potential probiotic functions.

### Restoration of microbiota-depleted probiotics alleviates chemotherapy-induced toxicity

To demonstrate the importance of microbiota-depleted probiotics in oxaliplatin-induced toxicity, we isolated three strains of probiotics (*Bifidobacterium longum, Lactobacillus reuteri*, and *Lactobacillus johnsonii*) from healthy volunteers and gavaged mice with this mixture after a 5-days treatment regimen of an antibiotic cocktail (Figure 6(e)). Importantly, treatment with these probiotics significantly alleviated weight loss and reduced the clinical score of toxicity in mice following chemotherapy exposure (Figures 6(f–g)). Overall survival was also significantly improved in mice treated with the probiotics mixture (Figure 6(h)). Histological analysis further revealed improved toxicity in the hematopoietic and gastrointestinal systems (Figures 6(i–k)). Moreover, the mRNA expression of pro-inflammatory molecules (IL-1β, IL-6, and TNF-α) was significantly decreased, whereas the expression of epithelial barrier molecules (claudin and occludin) was significantly increased in the colon (Figure S4(a)). TLR4 signaling-associated molecules were also significantly upregulated in splenocytes from probiotics-treated mice (Figure S4(b)). Notably, probiotics treatment significantly increased the percentage of F4/80^+^ IL-10^+^ macrophages in the spleen (Figure S4(c)). In addition, IHC results showed a significant increase in the proportion of macrophages and IL-10 expression in the colon, spleen, and bone marrow tissues from mice treated with probiotics (Figures 6(l-n)). Similar alleviation of chemotherapy-induced toxicity was also found in mice treated with a mixture of these three probiotics without antibiotic cocktail pre-treatment (Figures S4(d-f)). More importantly, significant improvement of chemotherapy-induced toxicity was further observed in tumor-bearing mice model after treatment of these three probiotics (Figures S4(g-n)). These findings indicate that supplementation with probiotics can improve chemotherapy-induced toxicity.

### Enrichment of short-chain fatty acids by probiotics improves chemotherapy-induced toxicity

Previous studies have demonstrated that probiotics favor the production of short-chain fatty acids (SCFAs).^17^ Butyrate engages dendritic cells and macrophages to promote IL-10 secretion.^18^ Next, we performed targeted metabolome analysis of the feces of mice administered probiotics. As expected, seven SCFAs, including butyrate, were significantly enriched in the mice treated with the probiotics mixture (Figure S5(a)). Additionally, flow cytometry analysis showed that probiotics supernatant increased the proportion of F4/80^+^ IL-10^+^ macrophages in *Rag1*^*-/-*^ mice (Figure S5(b)). To confirm the beneficial role of SCFAs in alleviating chemotherapy-induced toxicity, mice challenged with oxaliplatin were gavaged with butyrate or PBS (Figure S5(c)). Consistent with the above results, the weight loss and the clinical score of toxicity in mice treated with butyrate were significantly improved, as well as the toxicity of the hematopoietic and gastrointestinal systems (Figures S5(d–h)). Furthermore, IHC results showed a significant increase in the proportion of macrophages and IL-10 expression in the colon, spleen, and bone marrow tissues of mice treated with butyrate (Figures S5(i–k). These data suggest that the alleviation of chemotherapy-induced toxicity by probiotics is associated with the production of SCFAs.

### Improvement of toxicity does not influence efficacy of chemotherapy

**Figure 7.**
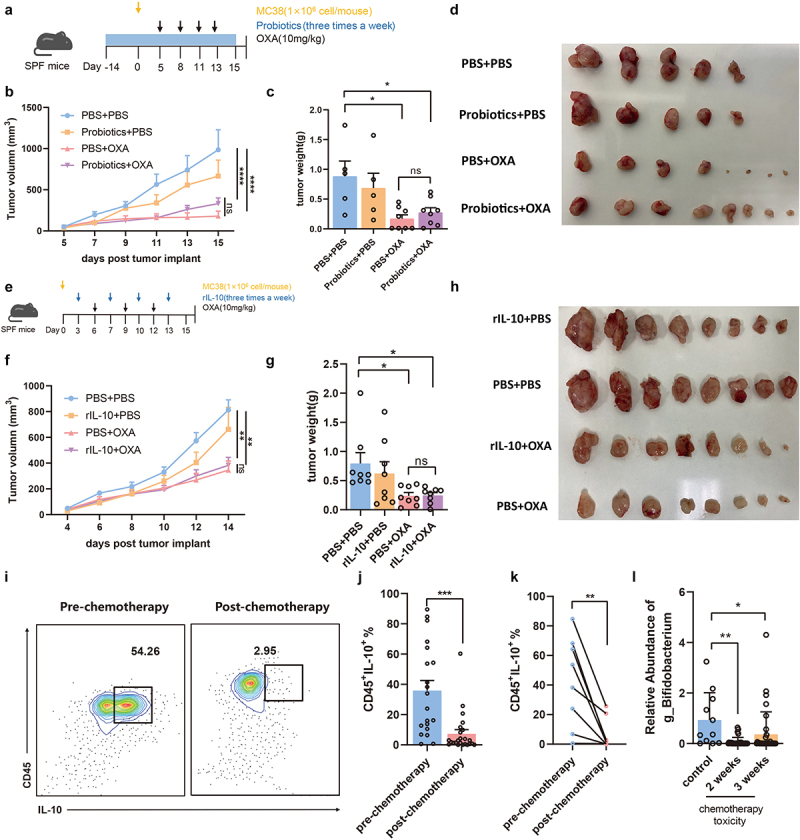
Improvement of toxicity does not influence the efficacy of chemotherapy. (a) Experimental design of supplement probiotics or PBS to SPF C57BL/6 mice with injection of MC38 cells, followed by oxaliplatin intervention. (b,c) Changes of tumor sizes (PBS+PBS vs. PBS+OXA: *p *< .0001, PBS+PBS vs. Probiotics+OXA: *p *< .0001, PBS+OXA vs. Probiotics+OXA: *p *= .3943) and tumor weights (PBS+PBS vs. PBS+OXA: *p *= .0160, PBS+PBS vs. Probiotics+OXA: *p *= .0464, PBS+OXA vs. Probiotics+OXA: *p *= .9439) in mice treated with probiotics or PBS. (d) Representative image of subcutaneous tumors from mice with treatment of probiotics or PBS. (e) Experimental design of supplement rIL-10 or PBS to SPF C57BL/6 mice with injection of MC38 cells, followed by oxaliplatin intervention. (f,g) Changes of tumor sizes (PBS+PBS vs. PBS+OXA: *p *= .0030, PBS+PBS vs. rIL-10+OXA: *p *= .0042, PBS+OXA vs. rIL-10+OXA: *p *= .9790) and tumor weights (PBS+PBS vs. PBS+OXA: *p *= .0452, PBS+PBS vs. rIL-10+OXA: *p *= .0491, PBS+OXA vs. rIL-10+OXA: *p *> .9999) in mice treated with rIL-10 or PBS. (h) Representative images of subcutaneous tumors from mice with treatment of rIL-10 or PBS. (i) Percentage of IL-10^+^CD45^+^ PBMCs from patients before and after chemotherapy detected by flow cytometry. (j) Changes of CD45^+^IL-10^+^ PBMCs from patients after chemotherapy treatment (*p *= .0001). (k) Changes of CD45^+^IL-10^+^ PBMCs from the same patient after chemotherapy treatment (*p *= .0067). (l)Relative abundance of the *Bifidobacterium* between the acute lymphoblastic leukemia children with intestinal toxicity and those sibling controls from a public microbiome dataset (control vs. 2 weeks: *p *= .0019, control vs. 3 weeks: *p *= .0431). Each dot indicates an individual. For j, pre-chemotherapy: *n*=20, post-chemotherapy: *n*=24. The statistical significance values are denoted as: *p < .05, **p < .01, ***p < .001, ****p < .0001. One-way ANOVA following Tukey’s multiple comparison test (c, g, and i); two-way ANOVA following Tukey’s multiple comparison test (b and f); two tailed student t test (j); paired Student t test (k).

To evaluate the efficacy of chemotherapy after amelioration of toxicity, we orally administered probiotics and intraperitoneally injected oxaliplatin into mice that subcutaneously harbored MC38 CRC cells (Figure 7(a)). Mice treated with chemotherapy exhibited slower tumor growth, as well as a corresponding reduction in tumor size and weight (Figures 7(b–d)). Notably, probiotics administration had no significant effect on the therapeutic efficacy of the chemotherapy. As an important cytokine in chemotherapy toxicity, rIL-10 injection in combination with oxaliplatin was subsequently administered to mice harboring subcutaneous MC38 cells (Figure 7(e)). Administration of IL-10 did not influence the efficacy of chemotherapy (Figures 7 (f-h)). These data indicate that the amelioration of chemotherapy-induced toxicity by probiotics or IL-10 does not influence the efficacy of chemotherapy.

To further verify the association between IL-10 and chemotherapy, we established a clinical cohort of CRC patients exposed to oxaliplatin neoadjuvant chemotherapy. The number of CD45^+^IL-10^+^ cells in the peripheral blood mononuclear cells (PBMCs) of CRC patients who did not receive chemotherapy was significantly higher than that in patients who received chemotherapy (Figures 7(i,j)). Importantly, CD45^+^IL-10^+^ cells in the peripheral blood of patients with CRC were significantly suppressed after treatment with chemotherapy (Figure 7(k)). Moreover, re-analysis of the public dataset PRJEB35526^8^ in children with acute lymphoblastic leukemia (ALL) showed a significant decrease in *Bifidobacterium* after two or three weeks of chemotherapy, which was associated with the occurrence of gastrointestinal toxicity (Figure 7(l)). These data observed in clinical cohorts confirmed an impaired IL-10 levels and a decrease in probiotics strains upon chemotherapy, suggesting potential new therapeutic targets for chemotherapy-induced toxicity.

## Discussion

hemotherapy-induced toxicity is an important impediment in cancer management. Understanding the underlying mechanisms responsible for this adverse effect will advance therapeutic research. Our data demonstrated that oxaliplatin-induced exacerbation of hematopoietic and gastrointestinal toxicity was caused by alterations in the intestinal microbiota, especially the depletion of beneficial taxa, such as *Bifidobacterium* and *Lactobacillus*. Furthermore, chemotherapy toxicity induced by the gut microbiota is dependent on decreased IL-10 secretion from macrophages. Targeted restoration of beneficial microbiota or IL-10 supplementation in mice improves oxaliplatin-induced toxicity through TLR4-mediated IL-10 production by macrophages. Importantly, targeted intervention to improve chemotherapy toxicity did not dampen the therapeutic efficacy of oxaliplatin against cancer in mice.

Understanding the impact of microbiota on chemotherapeutic-induced toxicity has been the subject of numerous studies. A study found that severe diarrhea caused by irinotecan was associated with an increased abundance of the cecal *Clostridium cluster XI* and *Enterobacteriaceae*, both of which are potentially pathogenic.^19^ Indeed, microbial-derived β-glucuronidase has been shown to actively contribute to irinotecan-induced toxicity in the gastrointestinal tract.^20^ The expression of β-glucuronidase has been found in several phyla, such as *Bacteroidetes*, *Firmicutes*, *Verrucomicrobia*, and *Proteobacteria* .^21^ More importantly, several genera including *Ruminococcus, Paramuribaculum and Clostridium* were found in mice exposed to high doses of oxaliplatin in our current study. And accumulation of gut *Ruminococcus* during chemotherapy may contribute to the development of gastrointestinal complications in ALL in children.^22^ In addition to the accumulation of pathogens, depletion of protective commensals is associated with gastrointestinal toxicity in acute lymphoblastic leukemia patients who received triple intrathecal therapy (prednisolone, methotrexate, and cytarabine).^8^ Consistently, our study also found the depletion of fecal *Lactobacillus* and *Bifidobacterium* in mice treated with high-dose oxaliplatin. These findings suggest that chemotherapeutic drugs may create a distinct gut microenvironment characterized by dysbiosis of deleterious and protective microbiota, thereby rendering patients susceptible to adverse effects that could be attenuated through microbial intervention.

Several studies have demonstrated that the efficacy of chemotherapy is driven by a microbiota-induced immune response.^23^ Cyclophosphamide was able to promote accumulation of Th17 and Th1-cell response through stimulation of gram-positive commensals.^24^ Activation of splenic effector CD4^+^ T cells and tumor-infiltrating lymphocytes by *Bacteroidales* was found to be correlated with the development of checkpoint-blockade-induced colitis and the efficacy of CTLA-4 blockade.^25,26^ Infiltration of tumor-specific T cells by anti-PD-L1 was also mediated by the enrichment of *Bifidobacterium*.^27^ However, the relationship between oxaliplatin-induced toxicity and the pattern of immune response remains unclear. Previous studies have shown that macrophages play an important role in capecitabine-induced hand-foot syndrome and chemotherapy-induced immunotoxicity.^13^ Our study demonstrated that oxaliplatin-induced toxicity was also macrophage-dependent via a mechanism involving impaired IL-10 secretion.

The current study demonstrated that IL-10 is produced by different subsets of leukocytes, including dendritic cells (DCs), macrophages, T cells, natural killer (NK) cells, and B cells.^28^ Specifically, it has been demonstrated that IL-10 secretion from macrophages was activated by the recognition of pathogen-derived products, highlighting the significant role of macrophage-derived IL-10 in response to the stimulation of microbiota.^29^ *Clostridium butyricum* induces the infiltration of IL-10-producing macrophages to suppress acute colitis in mice.^30^ A recent study also demonstrated that a combination of pegilodecakin (pegylated IL-10) and anti-PD-1 antibodies had preliminary antitumor activity in advanced solid tumors.^31^ Similarly, our study demonstrated that oxaliplatin-associated dysbiosis downregulated the secretion of IL-10 from macrophages, but not T or B lymphocytes. Importantly, supplementation of oxaliplatin-exposed mice with a probiotics cocktail (*Bifidobacterium longum, Lactobacillus reuteri*, and *Lactobacillus johnsonii*) attenuated toxicity, a phenotype associated with increased numbers of F4/80^+^IL-10^+^ macrophages. Interestingly, probiotics-gavaged mice showed an increased production of fecal-derived SCFAs, including butyrate, a microbial-derived metabolite known to increase IL-10 production in immune cells. These findings emphasize the role of microbiota in macrophage-derived IL-10 in controlling oxaliplatin-induced toxicity, thereby providing a novel therapeutic strategy for patients undergoing chemotherapy. Our clinical observation that patients with colorectal cancer exposed to oxaliplatin exhibited downregulation of peripheral CD45^+^IL-10^+^ cells reinforces the translational impact of our study.

IL-10 plays an important role in the regulation of host homeostasis. The association between IL-10 and intestinal injury has been demonstrated in several studies in both humans and animal models. For example, IL-10 suppresses small-intestinal inflammation and epithelial damage and prevents the infiltration of cytotoxic CD4^+^ intraepithelial lymphocytes.^32^ Spontaneous colitis in *Il10*^−/−^ mice is driven by IL-22 and implicates an under-appreciated IL-10/IL-22 axis in regulating intestinal homeostasis.^33^ The mechanism underlying the regulation of marrow suppression by IL-10 has also been reported in previous studies. IL-10-producing B cells in the bone marrow have been reported to be reduced in patients with aplastic anemia (AA) compared to healthy individuals, and IL-10-producing CD24^hi^CD38^hi^ Bregs reduced bone marrow failure.^34^ This possibility is also supported by evidence that IL-10 related DCs improved hematopoiesis and survival in an AA murine model, with decreased Th17 and increased Treg cells.^35,36^ Thus, these studies suggest a potential mechanism for IL-10 to alleviate chemotherapy-related toxicity in the hematopoietic and digestive systems.

Activation of macrophages by microorganisms is mediated by pattern recognition receptors (PRRs), which subsequently trigger the expression of cytokines and other factors.^28^ A previous study showed that oxaliplatin response was mediated by TLR4 and reactive oxygen species produced by myeloid cells.^37^ Moreover, TLR4 deficiency enhances intestinal damage and the severity of late-onset diarrhea following irinotecan-based treatment.^38^ Similarly, our present study demonstrated that secretion of IL-10 from macrophages was associated with the dysfunction of TLR4 and downstream NF-κB signaling pathway, leading to exacerbation of chemotherapy toxicity. Increasing studies have further demonstrated that therapeutic impact of probiotics on NF-κB signaling pathway was mediated the activation of TLR4 signaling pathway.^39^ This may be regulated by the induction of inducible nitric oxide synthase(iNOS) and nitric oxide (NO) production.^40^ And our current study further supported that supplement of probiotics rescued the downregulation of IL-10 in macrophages. In addition to the growth of probiotics, competitive exclusion of harmful bacteria by probiotics supplement may be another important mechanism to alleviate the chemotherapy-induced toxicity.^41^

This study highlights the role of microbiota in chemotherapy-induced toxicity and its underlying mechanisms. However, the present study had some limitations. Although a distinct pattern of the microbiome was found in mice treated with high-dose oxaliplatin, the microbiota profile in clinical cohorts needs to be assessed to determine physiological relevance. The mechanism how probiotics modulate the activation of TLR4 and downstream NF-κB signaling pathway in macrophages is far from clear. In addition, the therapeutic effect of probiotics on chemotherapy-induced toxicity in patients remains unclear and requires controlled clinical trials.

Treatment options for the adverse effects of chemotherapy are limited. Our work reveals a critical role for the microbiome in oxaliplatin-induced toxicity, which is mediated by the suppression of IL-10-producing macrophages. Targeting the microbiota by probiotics treatment could alleviate the toxicity of chemotherapy by restoring IL-10 secretion from macrophages. Therefore, elucidation of the role of microbiota and underlying mechanisms in chemotherapy toxicity provides a novel strategy for patients to improve chemotherapy tolerance and advance their therapeutic mission.

## Materials and methods

### Mice

Six-to eight-week-old male C57BL/6 and *IL-10*^*-/-*^, *Rag1*^*-/-*^, and *tlr4*^*Lps-del*^ mice were purchased from GemPharmatech. All mice were housed under a 12 h light-dark cycle in an SPF facility and fed a sterilized laboratory rodent diet, 5L0D (LabDiet).

### Bacterial strains

*Lactobacillus reuteri*, *Lactobacillus johnsonii*, and *Bifidobacterium longum* were isolated from healthy individuals and identified via 16S rRNA sequencing. All strains were grown at 37°C under anaerobic conditions in de Man, Rogosa, and Sharpe (MRS) medium.

### Human samples

Peripheral blood samples were collected before chemotherapy or after the fourth cycle of chemotherapy in CRC patients.

### Oxaliplatin intervention

A toxic dose of oxaliplatin (20 mg/kg body weight) was administered to mice via peritoneal injection. The mice were then housed in sterile autoclaved cages and provided standard chow and water ad libitum, unless otherwise noted. The mice were monitored for changes in body weight and other body parameters after the injection, unless otherwise noted. Clinical scores were determined using a cumulative scoring system (Supplementary Table S1), based on weight loss, temperature changes, physical appearance, posture, and mobility.^15^ Half of the serum from survived mice were used for the detection of routine blood parameters and another half of the serum from survival mice were used for the detection of oxaliplatin concentration. In the tumor-bearing mouse model, standard treatment doses (10 mg/kg body weight) or toxic doses (20 mg/kg body weight) of oxaliplatin were administered via peritoneal injection. The size and shape of the tumors were monitored every two days.

### Probiotics treatment experiment

All SPF C57BL/6 or *Il10*^*-/-*^ mice (male, 6–8 weeks old) were treated with a broad-spectrum antibiotic cocktail (ampicillin 0.2 g/L, metronidazole 0.2 g/L, neomycin 0.2 g/L, and vancomycin 0.1 g/L) in drinking water for five days. For probiotics colonization experiments, after a one-day washout period, mice were orally gavaged with a mixture of probiotics (1 × 10^9^ CFU/dose) or PBS thrice weekly, followed by oxaliplatin intervention.

### Liquid chromatograph mass spectrometer (LC-MS) analysis

For the serum samples, 50 µL samples were mixed with 300 µL mass spectrometry grade pre-chilled acetonitrile, then vortexed for 5 min. The mixture was then centrifuged at 15,000 × g and 4°C for 10 minutes, and the supernatant was collected. For fecal samples, 20 mg samples were weighed into a 2-mL screw top tube containing 50 mg of acid-washed glass beads, and then 120 µL mass spectrometry grade pre-chilled acetonitrile was added to each tube. The samples were homogenized under 70 Hz cryogenic grinding for 5 min. The tubes were then centrifuged at 15,000 × g and 4°C for 10 min, and the supernatant was collected. Measurements were obtained using an Agilent 1290 Infinity II Liquid Chromatography System coupled to an Agilent 6495A Triple Quadrupole Liquid Chromatography-Mass Spectrometry (LC-MS) System. Data analysis was conducted using MassHunter Workstation Data Acquisition, Agilent MassHunter VistaFlux Software, and Agilent Metabolite ID Software. The metabolites were identified based on the standards, MS/MS spectra, and the metabolite database METLIN (https://metlin.scripps.edu/indexphp).

### Fecal microbiota transplantation

SPF C57BL/6 donor mice were injected with oxaliplatin (20 mg/kg body weight) or PBS for two weeks. Fecal pellets (200–250 mg) were collected in sterile tubes prior to suspension and homogenization in 2 mL of PBS. After centrifugation at 100 × g at 4°C for 30 s, bacteria-enriched supernatants were collected and transplanted into mice (200 μL per mouse) by oral gavage three times weekly. Recipient mice were treated with an antibiotic cocktail for five days and a one-day washout period, followed by FMT intervention (three times a week).

### Macrophage depletion experiment

Mice were treated with an antibiotic cocktail for five days, after a one-day washout period, followed by intraperitoneal injection of clodronate liposomes or control liposomes (FormuMax) (200 μL per mouse) to eliminate macrophages. Subsequently, the FMT experiment was conducted for two weeks, as previously described. Mice were exposed to a high dose of oxaliplatin (20 mg/kg body weight).

### rIL-10 and SCFA treatment

Mice were intraperitoneally injected with rIL-10 (100 ng/mouse/injection in 0.1 mL of PBS; Novoprotein) twice a week during the course of oxaliplatin intervention. Sodium butyrate (200 mM) was administered to the mice in drinking water for two weeks, followed by oxaliplatin treatment.

### Macrophage isolation and adoptive transfer

Donor mice were sacrificed and the spleen was harvested. Spleen immune cell was isolated and macrophages were further isolated by using magnetic bead separation methods. In short, the cell number in the single cell suspension was determined and then centrifuged. Next, the cell pellet was incubated with anti-F4/80 microbeads (130-110-443, Miltenyi Biotec) according to the manufacturer’s instructions. Recipient mice were injected intravenously with 2 × 10^6^ macrophages. Then mice were treated with oxaliplatin after three days of injection.

### Tumor inoculation

Mice were subcutaneously inoculated with 10^6^ MC38 cells in the abdominal flank. Tumor volume was measured every two days and calculated using the formula (length × width^2^ ×0.5. Five days after tumor inoculation, oxaliplatin (10 mg/kg body weight) was administered to mice twice a week. For probiotic treatment, mice were gavaged with a mixture of probiotics (1 × 10^9^ CFU/dose) or PBS thrice weekly before tumor inoculation. For rIL-10 treatment, the mice were injected with rIL-10 twice before tumor inoculation.

### High throughput 16S rRNA amplicon sequencing and analysis

Genomic DNA was extracted using a FastDNA Spin Kit for Soil (MP Biomedicals). For 16S rRNA gene sequencing, the V3-V4 variable region was amplified using 2-step PCR. In the first step, 10 ng genomic DNA was used as a template for the first PCR with a total volume of 20 μl using the 338F (5’-ACTCCTACGGGAGGCAGCAG-3’) and 806 R (5’-GGACTACHVGGGTWTCTAAT-3’) primers appended with Illumina adaptor sequences. The amplicons were purified, checked on a fragment analyzer, quantified, followed by equimolar multiplexing, and sequenced on an Illumina MiSeq PE300 platform. The pooled amplicons were further qualified and quantified using the Microbial Ecology 2 (QIIME2) software. Reads were imported, quality-filtered, and dereplicated with the q2-data2 plugin. Subsequently, the dada2 plugin was used with paired-end reads, with truncation of the primer sequences and trimming of the reads. The sequences were classified using Greengenes2^42^ as the reference 16S rRNA gene database. PCoA, LEfSe, and significant species were analyzed using R (v4.1.1).^43^

### Host RNA sequencing and analysis

Splenic samples were obtained from mice subjected to FMT. Total RNA was extracted from splenic tissues using TRIzol Reagent (Invitrogen), according to the manufacturer’s instructions (Invitrogen). RNA integrity was evaluated using ND-2000 (NanoDrop Technologies, USA) and 2100 Bioanalyzer (Agilent Technologies). RNA-seq libraries were prepared using the TruSeq RNA Sample Prep kit (Illumina), and libraries were successfully constructed from splenic samples. Briefly, messenger RNA was isolated according to the polyA selection method using oligo (dT) beads, and then fragmented using fragmentation buffer. Double-stranded cDNA was synthesized using a SuperScript double-stranded cDNA synthesis kit (Invitrogen) with random hexamer primers (Illumina). Then the synthesized cDNA was subjected to end-repair, phosphorylation and ‘A’ base addition according to Illumina’s library construction protocol. Libraries were selected for cDNA target fragments of 300 bp on 2% low range ultra-agarose, followed by PCR amplification using Phusion DNA polymerase (NEB) for 15 PCR cycles. After quantification using TBS380, the paired-end RNA-seq sequencing library was sequenced using the Illumina HiSeq xten/NovaSeq 6000 sequencer (2 × 150bp read length). Differential expression analysis between the two groups was performed using the Limma R package. Genes with an adjusted *p*-value <0.05 and |Log2(Fold Change)|＞0 were assigned as significantly differentially expressed.

### Measurement of serum cytokine levels using multiplex immunoassays

A total of 31 serum cytokines were detected simultaneously using the Bio-Plex Pro Human Cytokine Screening Panel (R&D Systems), according to the manufacturer’s protocol. The tests were performed in accordance with the manufacturer’s procedures, and the sample dilution was 1:2, including the standard curve and blank value. The assay plate was analyzed using a Luminex X-200instrument (Bio-Rad Laboratories). Data were calculated using the Bio-Plex Manager software ver. 5.0 (Bio-Rad Laboratories).

### Targeted metabolome of fecal SCFAs

SCFAs were extracted from fecal samples (100 mg) in an aqueous solution and analyzed by gas chromatography-mass spectrometry (GC-MS) using an Agilent 7890A/5975C instrument (BioNovoGene Company). Chromatographic separation was performed on an Agilent HP-5 capillary column. The analytes were quantified using a series of stock solutions under standard conditions. Briefly, 100 mg of fecal samples were weighed and mixed with 1 mL of 0.005 M NaOH solution with 50 μL 2-methyl-butyric acid for 2 min and incubated at 4°C for 2 h. Next, the mixture was centrifuged at 4°C 13,000 rpm for 20 min, and the supernatant was collected. A total of 500 μL of supernatant was added to 300 μL distilled water, 500 μL isopropanol/pyridine solution, and platelet cytotoxic factor solution for derivatization and then extracted with 500 μL n-hexane for further analysis. Agilent HP-5 capillary column (30 cm *0.25 mm *0.25 μm) was used for GC-MS detection. An Agilent MSD ChemStation (E.02.00.493, Agilent Technologies) was used to analyze the data.

### Cell isolation of spleen mononuclear cells

Spleens were completely isolated from mice and crushed with forceps, and single cells were isolated in PBS using a 70-μm cell strainer. The cells were washed with 1× PBS and centrifuged (100 × g for 5 min), and then red blood cell lysis containing splenocytes was pipetted up. The culture medium was then added to the cells and centrifuged at 100 × g at 4°C for 5 min. Single-cell suspensions were diluted in Roswell Park Memorial Institute (RPMI) medium.

### Isolation of BMDMs and PBMCs

BMDMs were isolated from the femurs and tibias of mice. Cells were differentiated in BMDM media (Dulbecco’s modified Eagle’s medium (DMEM), 10% fetal bovine serum (FBS), 25 mM l-glutamate, penicillin/streptomycin, and 200 U/mL recombinant mouse M-CSF. On day 7, the cells were challenged with fecal supernatants and allowed to acclimatize for 24 h.

Approximately 4 mL of human venous blood was collected in heparinized vials and gently inverted. PBMCs were isolated by gradient centrifugation using Ficoll-paque plus (Cytiva). Isolated cells were washed twice with 10 mL sterile FBS-free Roswell Park Memorial Institute (PRMI) medium. The medium was discarded, and the cells were resuspended in sterile PRMI medium.

### Cell culture and cellular stimulation

The murine macrophage cell line RAW264.7 was purchased from the American Type Culture Collection and cultured at 37°C in DMEM (Gibco) supplemented with 10% FBS (Gibco) in a 5% CO_2_ atmosphere.

For fecal supernatant stimulation experiments, a ratio of 1 mL PBS per 50 mg feces was used for homogeneously making fecal suspensions, centrifuged at 100 × g for 5 min, and the supernatant was collected. The supernatant was passed through a needle filter to remove the microbiota. The cells (RAW264.7, BMDM, and splenocytes) were stimulated by fecal supernatant for 24 h and RNA was extracted for qPCR analysis or cells were collected for flow cytometry.

### RNA and DNA extraction for qPCR analysis

RNA was extracted using a Total RNA Kit (R323–01; Vazyme). cDNA was reverse transcribed using Hiscript@ III RT Super Mix with a gDNA wiper (R323–01, Vazyme). Fecal or bacterial DNA was obtained using an AmPure Microbial DNA Kit (D7111, Megan). qPCR was performed on an Applied Biosystems 7500 Real-Time PCR system using SYBR Green real-time PCR master mix (QPK-201; Toyobo). The primer sequences used in this study are listed in Supplementary Table S2.

### Histopathology

Spleens, femurs, and colon tissues were collected, fixed in 10% neutral buffered formalin, embedded in paraffin, and sectioned. Before paraffin embedding, femurs underwent an additional decalcification step. The slides were stained with hematoxylin and eosin and morphological changes were observed.

### Flow cytometry analysis

For IL-10 detection, splenocytes were stimulated with PMA, ionomycin, and brefeldin A for 6 h. PBMCs were stimulated with LPS and brefeldin A for 24 h. The cells were resuspended in PBS and Fc receptors were blocked with anti-CD16/32 antibody (#101320, BioLegend). The following antibodies were used: CD4 (#100411, BioLegend), CD11b (#101226, BioLegend), F4/80 (#123122, BioLegend), and CD45 (#304006, BioLegend). For the intracellular marker IL-10 (#505008 and #501404, BioLegend), FOXP3 (#126405, BioLegend) cells were fixed and permeabilized using the FOXP3/Transcription Factor Staining Buffer kit (#00-5523-00, eBioscience) according to the manufacturer’s instructions after surface staining and incubated with the corresponding antibodies. Labeled cells were analyzed using a CytoFLEX flow cytometer (BECKMAN). Gating strategies are shown in Figure S6.

### Immunohistochemical staining

IL-10, F4/80, Foxp3, and CD4 expression in the spleen, femur, and colon tissues was determined by IHC. In brief, the following steps were performed: paraffin sections dewaxing to water; the antigen was retrieved; the endogenous peroxidase was blocked with hydrogen peroxide solution; 3%BSA was added at room temperature for 30 min; Anti-IL-10 (GB11108–100, Servicebio) antibody (1:500), Anti -F4/80 (GB113373–100, Servicebio) antibody (1:500), Anti -FOXP3 (GB112325–100, Servicebio) antibody (1:500), or Anti-CD4 (GB15064–100, Servicebio) antibody (1:500) was added, and the mix was refrigerated at 4°C overnight; sheep anti-mouse/rabbit IgG was added; we performed staining with diaminobenzidine (DAB) for 5 min and counterstaining with hematoxylin; the slices were then dehydrated and sealed with neutral gum.

The evaluation criteria for IHC experiments were as follows: The IHC results were obtained in a blinded manner (ImageJ Software) using an established pathological scoring system (H-score), and the intensity of staining was scored as 0= negative, 1= weak, 2= moderate, or 3= strong, and for each intensity, the frequency was indicated in percent (in steps of 10). The H-score was then calculated as the sum of 1× frequency of weak staining + 2× frequency of moderate staining + 3× frequency of strong staining.

### Quantification and statistical analysis

All data are expressed as mean ± SEM unless otherwise stated in the figure legends. Unless otherwise stated in individual method sections above, all statistical analyses were performed using Prism 8 (GraphPad Software). Two-tailed Student’s t-test (parametric) or Mann – Whitney U test (non-parametric) was used. For comparison of more than three groups, statistical analysis was performed using one-way ANOVA (parametric) or Kruskal-Wallis test (non-parametric). All *p*-values were two-sided, and an adjusted *p*-value of < 0.05 was considered statistically significant. The details of the statistical tests used and the pooled values for several biological replicates are indicated in the respective figure legends. Statistically significant values are denoted as *p < .05, **p < .01, ***p < .001, and ****p < .0001.

## Supplementary Material

Supplemental Material

## Data Availability

The sequencing data used in our manuscript has been uploaded. The 16S rRNA gene sequence data are available at the NCBI by accession number PRJNA902737. The RNA-seq data files have been deposited in NCBI’s BioProject under accession number PRJNA903109. Public datasets are available at the NCBI by accession number PRJEB35526.
