# Supplementary material for: Chemotherapy-induced microbiota exacerbates the toxicity of chemotherapy through the suppression of interleukin-10 from macrophages
Source: Gut Microbes. 2024 Feb 24;16(1):2319511. doi: 10.1080/19490976.2024.2319511 (PMC10896127; doi:10.1080/19490976.2024.2319511)
Supplement: Supplemental Material [file KGMI_A_2319511_SM4076.zip › KGMI_A_2319511_supplemental material/Extended data clean.docx]

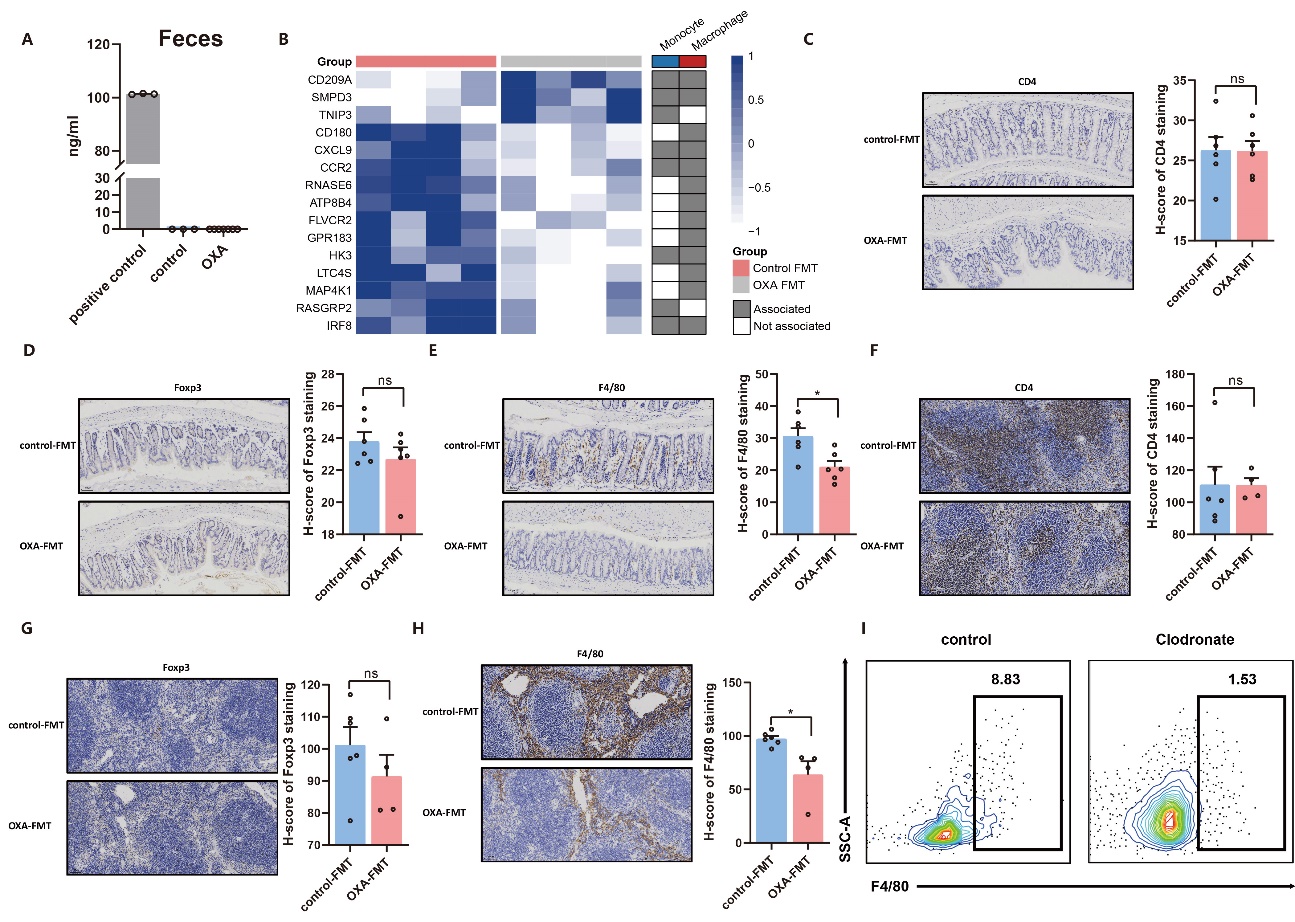
Extended data

**Figure S1 Microbiota-mediated toxicity of chemotherapy is macrophage-dependent. (A)** Concentration of oxaliplatin in feces by LC-MS. (Positive control: n=3; control: n=3; OXA: n=7). **(B)** Heatmap of the differential genes associated with monocytes and M2 macrophages. **(C-E)** In colon tissue, the immunohistochemical staining of CD4 (*p*=0.9742), Foxp3 (*p*=0.2637), and F4/80 (*p*=0.0124) was analyzed from the perspective of histological grades (H score). **(F-H)** In spleen tissue, the immunohistochemical staining of CD4 (*p*=0.9768), Foxp3 (*p*=0.3016), and F4/80 (*p*=0.0123) was analyzed from the perspective of histological grades (H score). **(I)** The proportion of macrophages in clodronate liposomal or PBS liposomal treated mice were analyzed by flow cytometry. Each dot indicates an individual mouse. The statistical significance values are denoted as: * *p*<0.05. Two tailed Student t test (**C-H**).

**
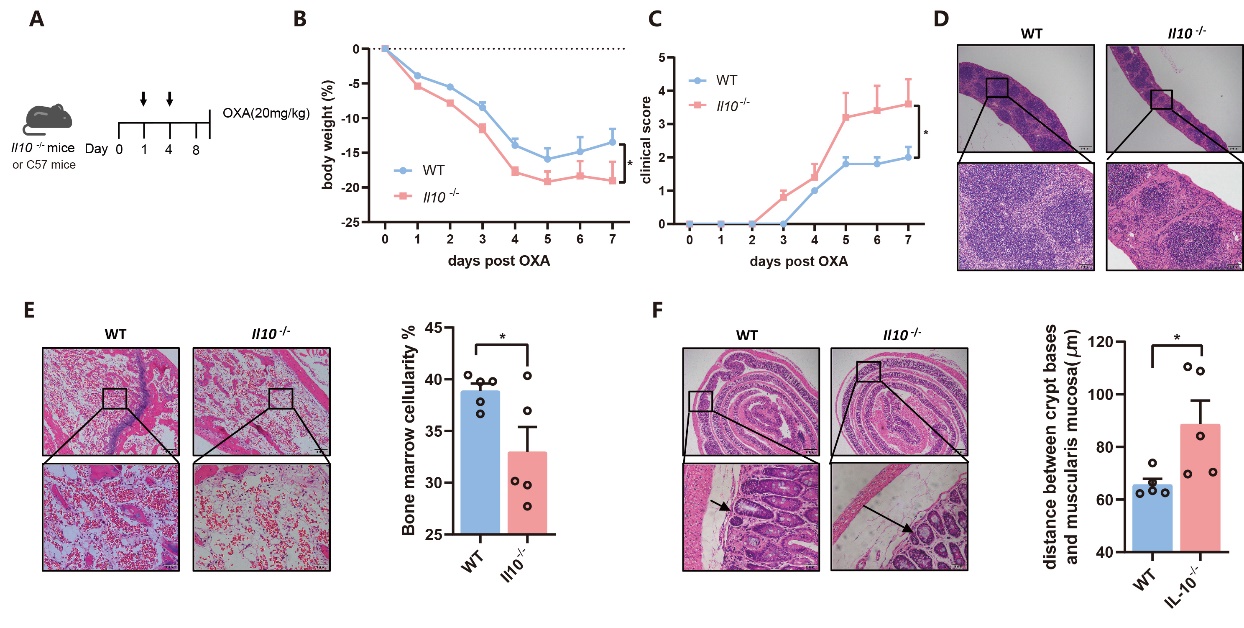
Figure S2 *Il10^-^*^/-^ mice exhibit severe toxicity from chemotherapy. (A)** Experimental schematic of oxaliplatin-exposed mice. **(B-C)** Changes of body weight (*p*=0.0336) **(B)** and clinicalscore (*p*=0.0197) **(C)** after administration of oxaliplatin. **(D)** Histopathological images of spleen. **(E)** Femurs were stained with H&E and quantification for bone marrow cellularity (*p*=0.0467). **(F)** Histopathological images of colon and quantification for the gaps between crypt bases and muscularis mucosa (*p*=0.0369). Arrows indicate gaps between crypt bases and muscularis mucosa. Each dot indicates an individual mouse. For **B-C**, WT: n=5, *Il10^-/-^*: n=5. The statistical significance values are denoted as: * *p*<0.05, ** *p*<0.01, *** *p*<0.001, **** *p*<0.0001. Two-way ANOVA following Sidak's multiple comparison test (**B** and **C**); two tailed Student t test (**E** and **F**).

**
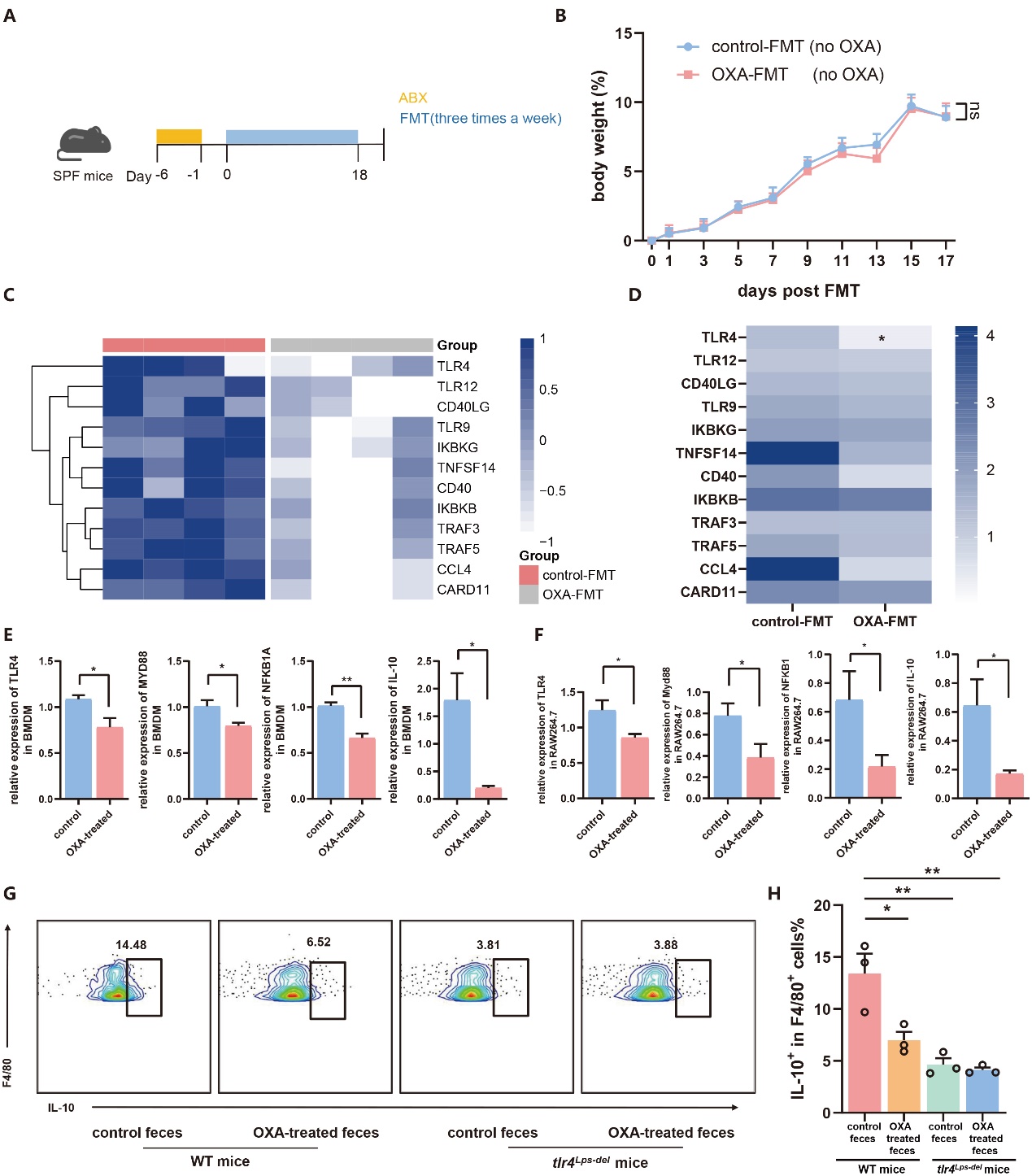
Figure S3** **Suppression of IL-10 in macrophages is responsible for chemotherapy-induced toxicity. (A)** FMT experimental design. **(B)** Changes of body weight (*p* >0.9999). **(C)** Heatmap of the differential genes associated with toll-like receptor signaling pathway and NF-κB signaling pathway. **(D)** mRNA level of genes in spleen validated by qPCR. **(E)** Relative mRNA levels of TLR4 (*p*=0. 0.0478), Myd88 (*p*=0.0291), NFKB1A (*p*=0.0014), and IL-10 (*p*=0.0177) in BMDMs from B57BL/6 mice after culture with feces supernatant from control mice or oxaliplatin treated mice for 24 hours.**（F）**Relative mRNA levels of TLR4 (*p*=0.0206), Myd88 (*p*=0. 0.0437), NFKB1A (*p*=0.0461), and IL-10 (*p*=0.0267) in RAW264.7 macrophage cell lines after culture with feces supernatant of control mice or oxaliplatin treated mice for 24 hours. **(G)** Splenocytes from *tlr4^Lps-del^* mice or WT mice were cultured with feces supernatant from control mice or oxaliplatin treated mice for 24 hours. Expression of IL-10 in F4/80^+^ macrophages from *tlr4^Lps-del^* mice or WT mice were analyzed by flow cytometry. **(H)** Statistical analysis of the percentages of IL-10^+^ F4/80^+^ cells in mice spleen (WT control feces vs. WT OXA-treated feces: *p*= 0.0124, WT control feces vs. *tlr4^Lps-del^* control feces: *p*= 0.0023, WT control feces vs. *tlr4^Lps-del^* OXA-treated feces: *p*= 0.0019). Each dot indicates an individual mouse. For **B**, control-FMT (no OXA): n=9, OXA-FMT (no OXA): n=9. The statistical significance values are denoted as: * *p*<0.05. Two-way ANOVA following Sidak's multiple comparison test (**B**); one-way ANOVA following Tukey’s multiple comparison test (**H**); two tailed Student t test (**D-F**).

**
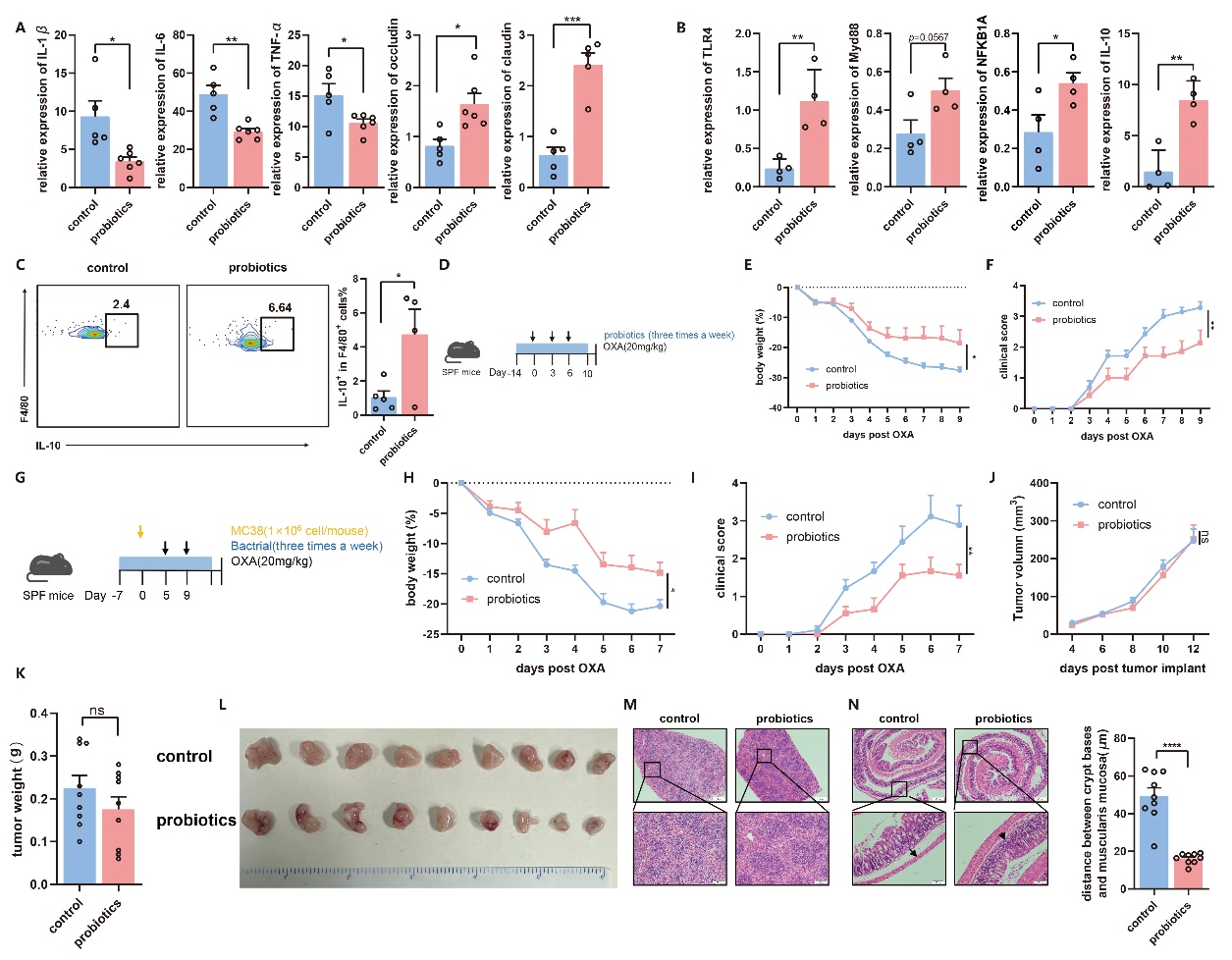
Figure S4 Probiotics administration alleviate oxaliplatin-induced toxicity in mice. (A)** Relative mRNA levels of IL-1β (*p*=0.0144), IL-6 (*p*=0.0020), TNF-α (*p*=0.0330), occluding (*p*=0.0120) and claudin (*p*=0.0002) in the colon. **(B)** Relative mRNA levels of TLR4 (*p*=0.0066), Myd88 (*p*=0.0567), NFKB1A (*p*=0.0297) and IL-10 (*p*=0.0025) in the splenocytes **(C)** Percentages of IL-10^+^ F4/80^+^ cells in spleen from mice treated with probiotics or PBS (*p*=0.0310). **(D)** Experimental design of probiotic administration and oxaliplatin intervention in SPF C57BL/6 mice . **(E-F)** Changes of body weight (*p*=0.0260) **(E)** and clinical score (*p*=0.0025) **(F)** after administration of oxaliplatin. **(G)** Experimental design of probiotic administration and oxaliplatin intervention in SPF C57BL/6 mice with injection of MC38 cells. **(H-I)** Changes of body weight (*p=*0.0366) **(H)** and clinical score (*p*=0.0095) **(I)** after administration of oxaliplatin. **(J-K)** Changes of tumor sizes (*p*>0.9999) and tumor weights (*p*= 0.2612) in mice treated with probiotics or control. **(L)** Representative images of subcutaneous tumors from mice with treatment of probiotics or control. **(M)** Rrepresentative histopathological images of spleen. **(N)** Rrepresentative histopathological images of colon and quantification for the gaps between crypt bases and muscularis mucosa (*p*<0.0001). Each dot indicates an individual mouse. For **D**, control: n=7, probiotics: n=7. For **G**, control: n=9, probiotics: n=9. The statistical significance values are denoted as: * *p*<0.05, ** *p*<0.01, *** *p*<0.001, **** *p*<0.0001. Two-way ANOVA following Sidak's multiple comparison test (**E**, **F, H, I and J**); two tailed Student t test (**A**, **B**, **C, K and N**).

**
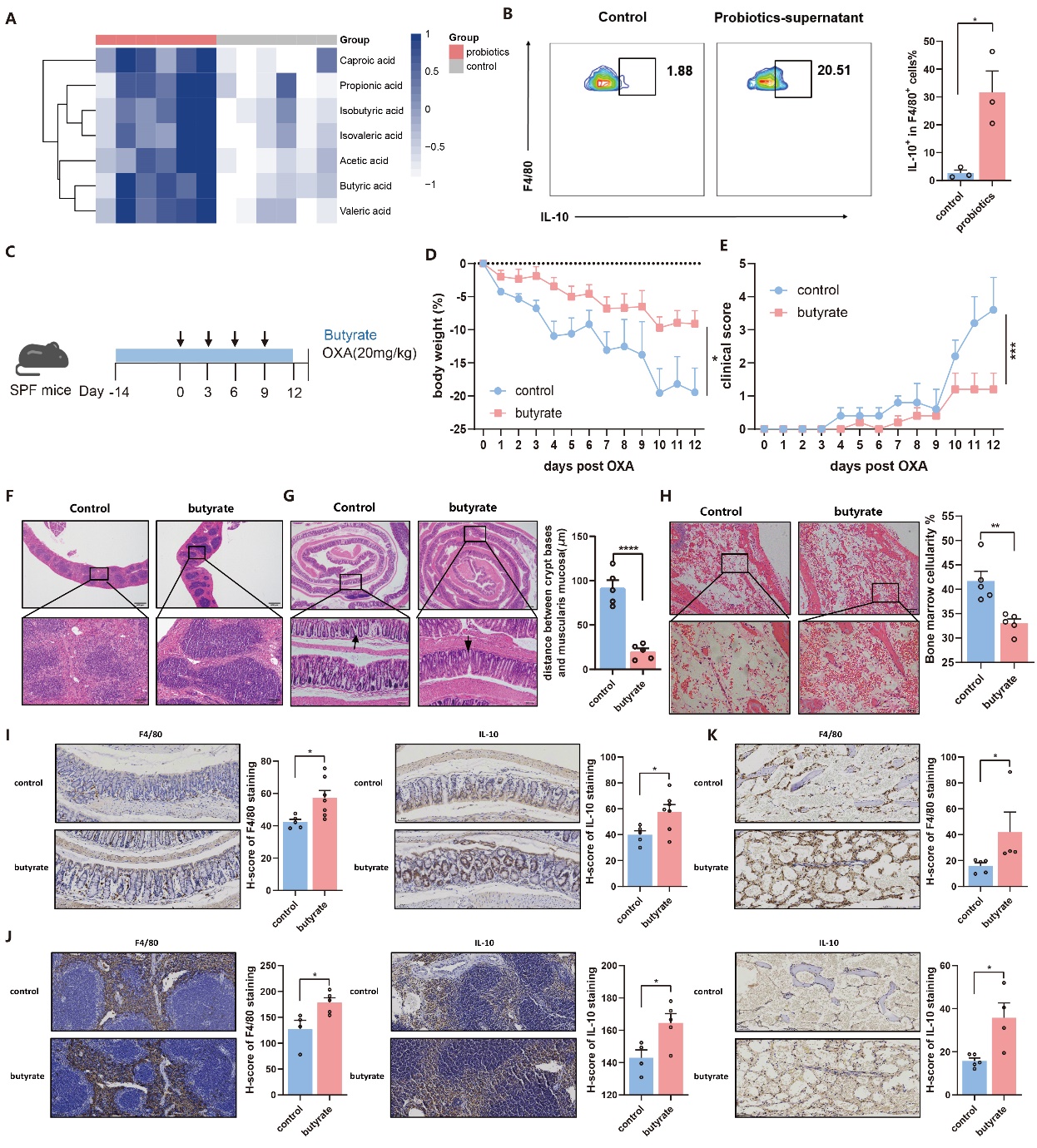
Figure S5 Fecal supernatant from probiotic-gavaged mice or butyrate administration alleviates chemotherapy-induced toxicity. (A)** Targeted metabolome of feces from mice gavaged probiotics or PBS. **(B)** Splenocytes from *Rag1^-/-^* mice were exposed to culture supernatant from control or probiotics for 24 hours. Expression of IL-10 in F4/80^+^ macrophages were analyzed by flow cytometry (*p*= 0.0198). **(C)** Experimental design for butyrate administration and oxaliplatin intevention. **(D-E)** Changes of body weight (*p*= 0.0323) **(D)** and clinical score (*p*= 0.0002) **(E)** after administration of oxaliplatin. **(F)** Histopathological images of spleen. **(G)** Histopathological images of colon and quantified for the gaps between crypt bases and muscularis mucosa (*p*<0.0001). Arrows indicate gaps between crypt bases and muscularis mucosa. **(H)** Femurs were stained with H&E and quantification for bone marrow cellularity (*p*=0.0043). **(I)** In colon tissue, the immunohistochemical staining of F4/80 (*p*= 0.0233), and IL-10 (*p*= 0.0302) was analyzed from the perspective of histological grades (H score). **(J)** In spleen tissue, the immunohistochemical staining of F4/80 (*p*= 0.0262), and IL-10 (*p*= 0.0277) was analyzed from the perspective of histological grades (H score). **(K)** In femur tissue, the immunohistochemical staining of F4/80 (*p*= 0.0159), and IL-10 (*p*= 0.0159) was analyzed from the perspective of histological grades (H score). Each dot indicates an individual mouse. For **C-E**, control: n=5, butyrate, n=5. The statistical significance values are denoted as: * *p*<0.05, ** *p*<0.01, *** *p*<0.001, **** *p*<0.0001. Two-way ANOVA following Sidak's multiple comparison test (**D** and **E**); two tailed Student t test (**B**, **G**, **H-K**).


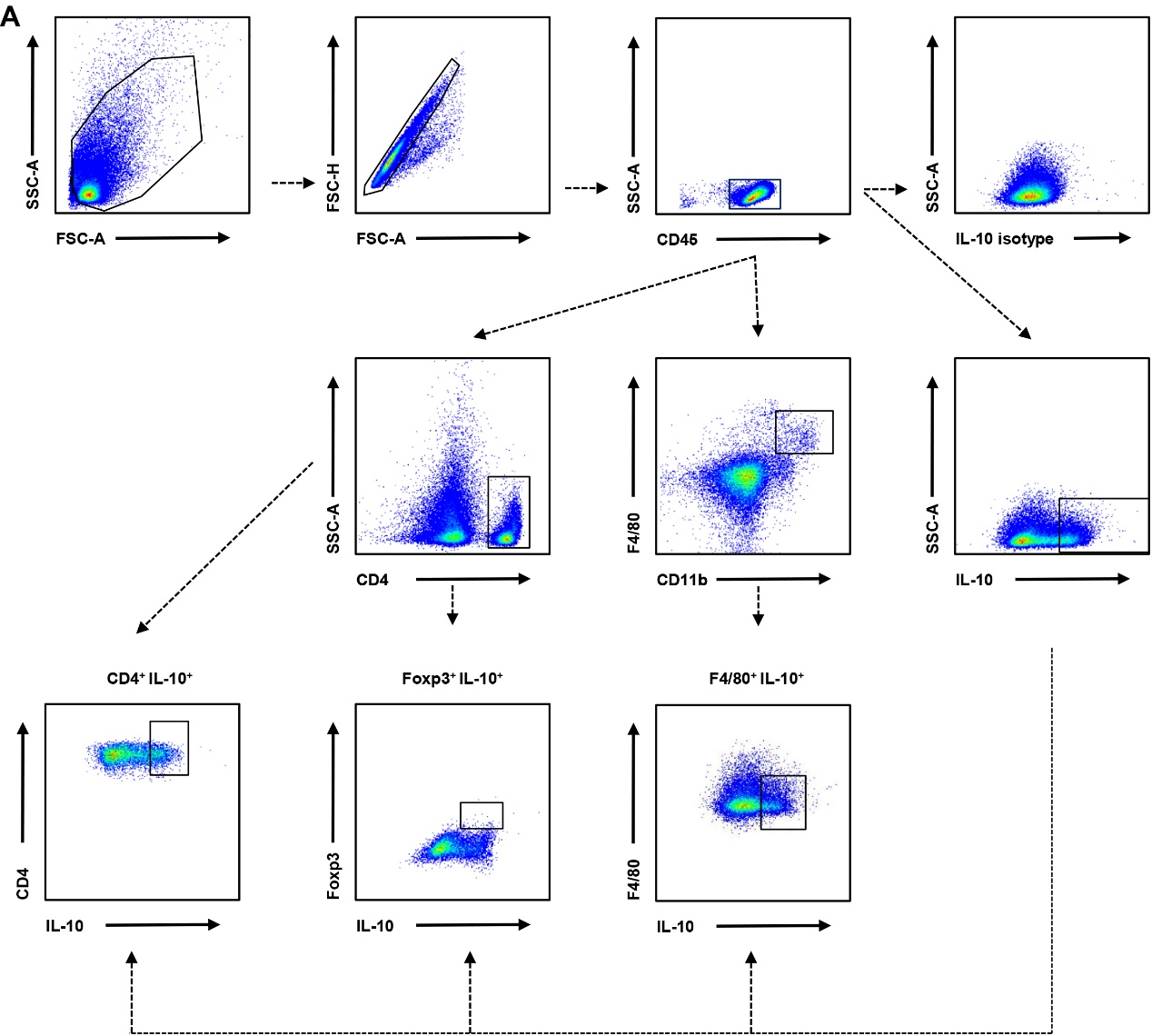


**Figure S6 Gating strategies for different immune cell populations. (A)** Gating strategies for IL-10^+^ macrophages (CD45^+^CD11b^+^F4/80^+^IL-10^+^), IL-10^+^CD4^+^ T cells (CD45+ CD4^+^IL-10^+^), and IL-10^+^ Treg cells (CD45^+^ CD4^+^Foxp3^+^ IL-10^+^).

**Table S1. Parameters used for the clinical score determination of mice.**

| **score** | | **0** | **1** | **2** |
| --- | --- | --- | --- | --- |
| Appearance | Weight loss | < 10% | > 10% to < 25% | > 25% |
|  | Coat | Normal | Mild ruffled coat | Moderate ruffled coat,  ungroomed |
|  | Body  condition | Normal | Thin | Loss of body fat, failure to grow |
|  | Body  Posture | Normal | Hunched | Hunched and still |
|  | Movement | Normal | Reduced/slow | Reluctant to move when touched |
| Activity | Proximity to  others | Close  contact | Somewhat separate | Completely separate |
| Other | Injection site | Normal | Some redness at  margins | Redness and swelling |

**Table S2. Primer sequences used in this study.**

| **Origin** | **Primers** | **Forward** | **Reverse** |
| --- | --- | --- | --- |
| **Mouse** | **IL-1β** | GCTGAAAGCTCTCCACCTCA | GCTTGGGATCCACACTCTCC |
|  | **IL-6** | CTCTGCAAGAGACTTCCATCCA | GACAGGTCTGTTGGGAGTGG |
|  | **TNF-⍺** | GCCTCTTCTCATTCCTGCTTG | CTGATGAGGGAGGCCATT |
|  | **TLR4** | ATGGCATGGCTTACACCACC | GAGGCCAATTTTGTCTCCACA |
|  | **Myd88** | TCATGTTCTCCATACCCTTGGT | AAACTGCGAGTGGGGTCAG |
|  | **NFKB1A** | TGCGATTCCGCTATAAATGCG | ACAAGTTCATGTGGATGAGG |
|  | **IL-10** | GCCAGTACAGCCGGGAAGACAATA | GCCTTGTAGACACCTTGGTCTT |
|  | **ZO-1** | TCATCCCAAATAAGAACAGAGC | GAAGAACAACCCTTTCATAAGC |
|  | **Occludin** | CTTTGGCTACGGAGGTGGCTAT | CTTTGGCTGCTCTTGGGTCTG |
|  | **Claudin** | GCTGGGTTTCATCCTGGCTTCT | CCTGAGCGGTCACGATGTTGTC |
|  | **Gapdh** | TGAAGCAGGCATCTGAGGG | CGAAGGTGGAAGAGTGGGAG |
|  | **TLR12** | CCTGGTCTCCCGCTATTTCAC | CCGAGGTACAACTTCCAAGGT |
|  | **CD40LG** | CCTTGCTGAACTGTGAGGAGA | CTTCGCTTACAACGTGTGCT |
|  | **TLR9** | ATGGTTCTCCGTCGAAGGACT | GAGGCTTCAGCTCACAGGG |
|  | **IKBGK** | AAGCACCCCTGGAAGAACC | TCTCAGGAGTACCCTGCTCTG |
|  | **TNFSF14** | ACTGCATCAACGTCTTGGAGA | TGGCTCCTGTAAGATGTGCTG |
|  | **CD40** | TGTCATCTGTGAAAAGGTGGTC | ACTGGAGCAGCGGTGTTATG |
|  | **IKBKB** | ATCAGGCGACAGGTGAACAG | GGCCACAGCAGTTCTCGAA |
|  | **TRAF3** | CAGCCTAACCCACCCCTAAAG | CGGCACTTCTCGCACTTGT |
|  | **TRAF5** | CACTCCGTGCTTCACAACC | GCGTTTTTGCAGTAGACGTGTA |
|  | **CCL4** | TTCCTGCTGTTTCTCTTACACCT | CTGTCTGCCTCTTTTGGTCAG |
|  | **CARD11** | GGAGGGCCAGCTATGGATGA | CCGGTTGCATTCCACGTTA |
| ***Bifidobacteria*** | | GATTCTGGCTCAGGATGAACGC | CTGATAGGACGCGACCCCAT |
| ***Lactobacillus*** | | AGCAGTAGGGAATCTTCCA | CACCGCTACACATGGAG |
