# Supplementary figures and images for: Chemotherapy-induced microbiota exacerbates the toxicity of chemotherapy through the suppression of interleukin-10 from macrophages
Source: Gut Microbes. 2024 Feb 24;16(1):2319511. doi: 10.1080/19490976.2024.2319511 (PMC10896127; doi:10.1080/19490976.2024.2319511)

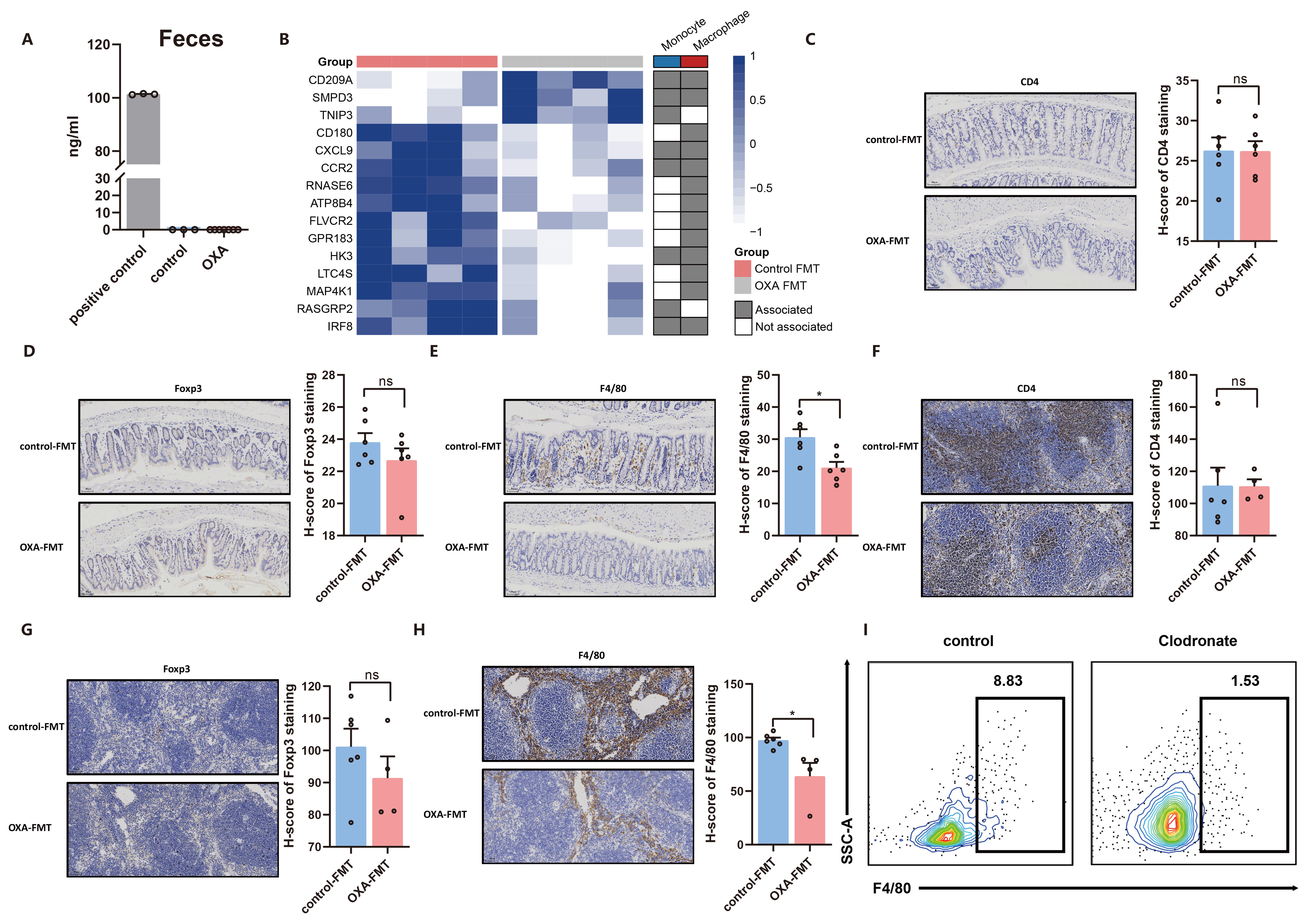

Supplement: Supplemental Material [file KGMI_A_2319511_SM4076.zip › KGMI_A_2319511_supplemental material/figure s1.jpg]

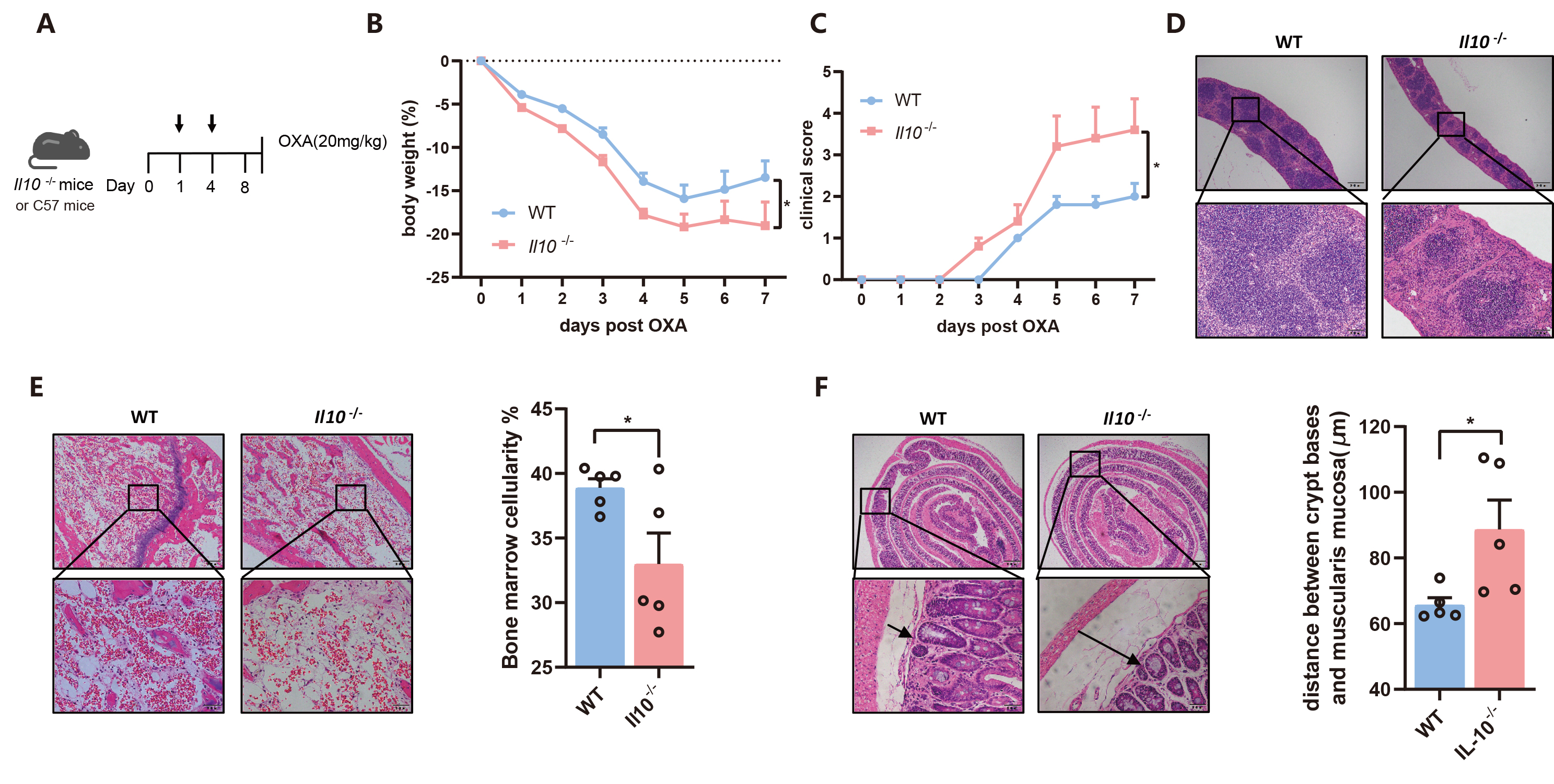

Supplement: Supplemental Material [file KGMI_A_2319511_SM4076.zip › KGMI_A_2319511_supplemental material/figure s2.jpg]

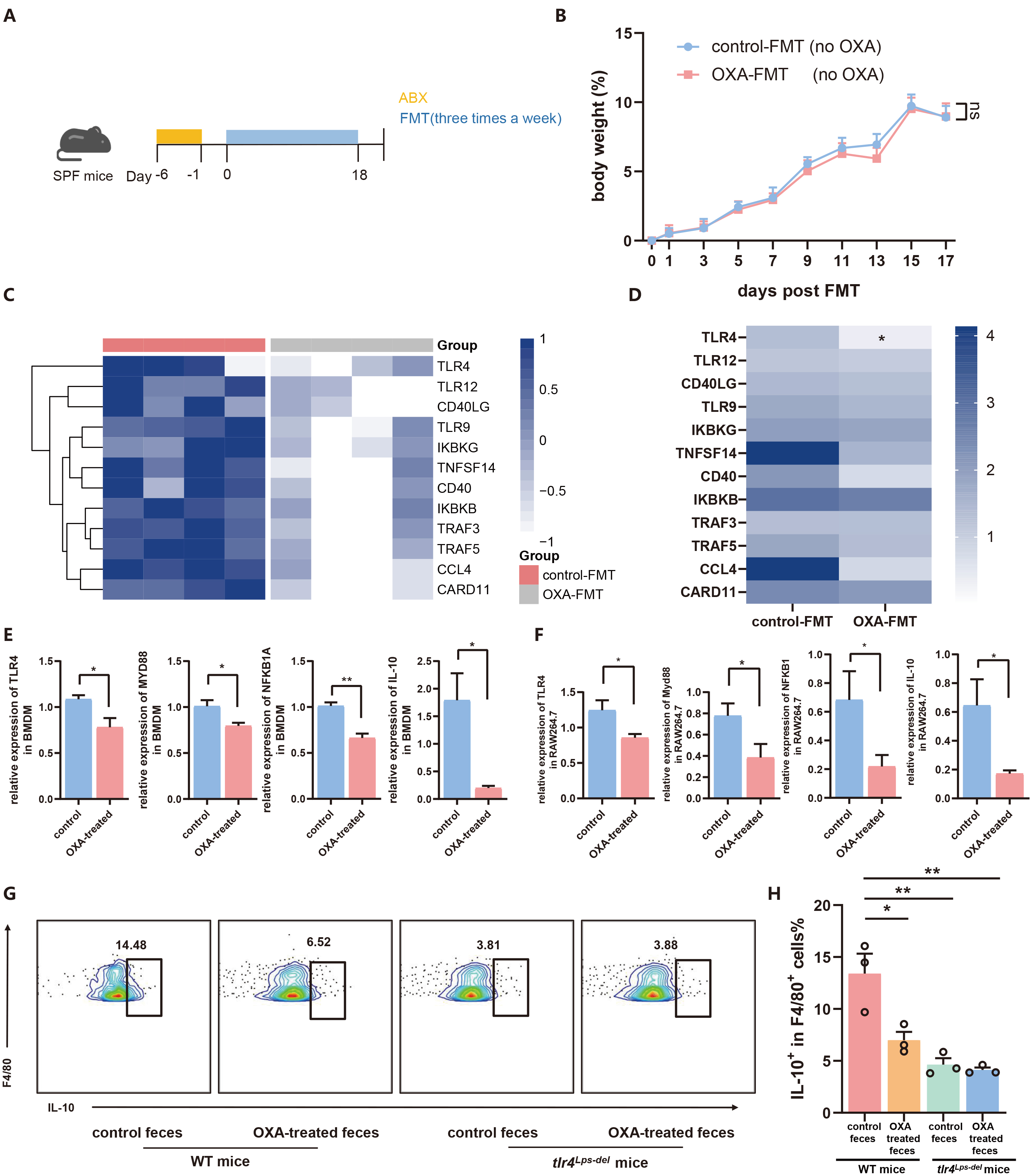

Supplement: Supplemental Material [file KGMI_A_2319511_SM4076.zip › KGMI_A_2319511_supplemental material/figure s3.jpg]

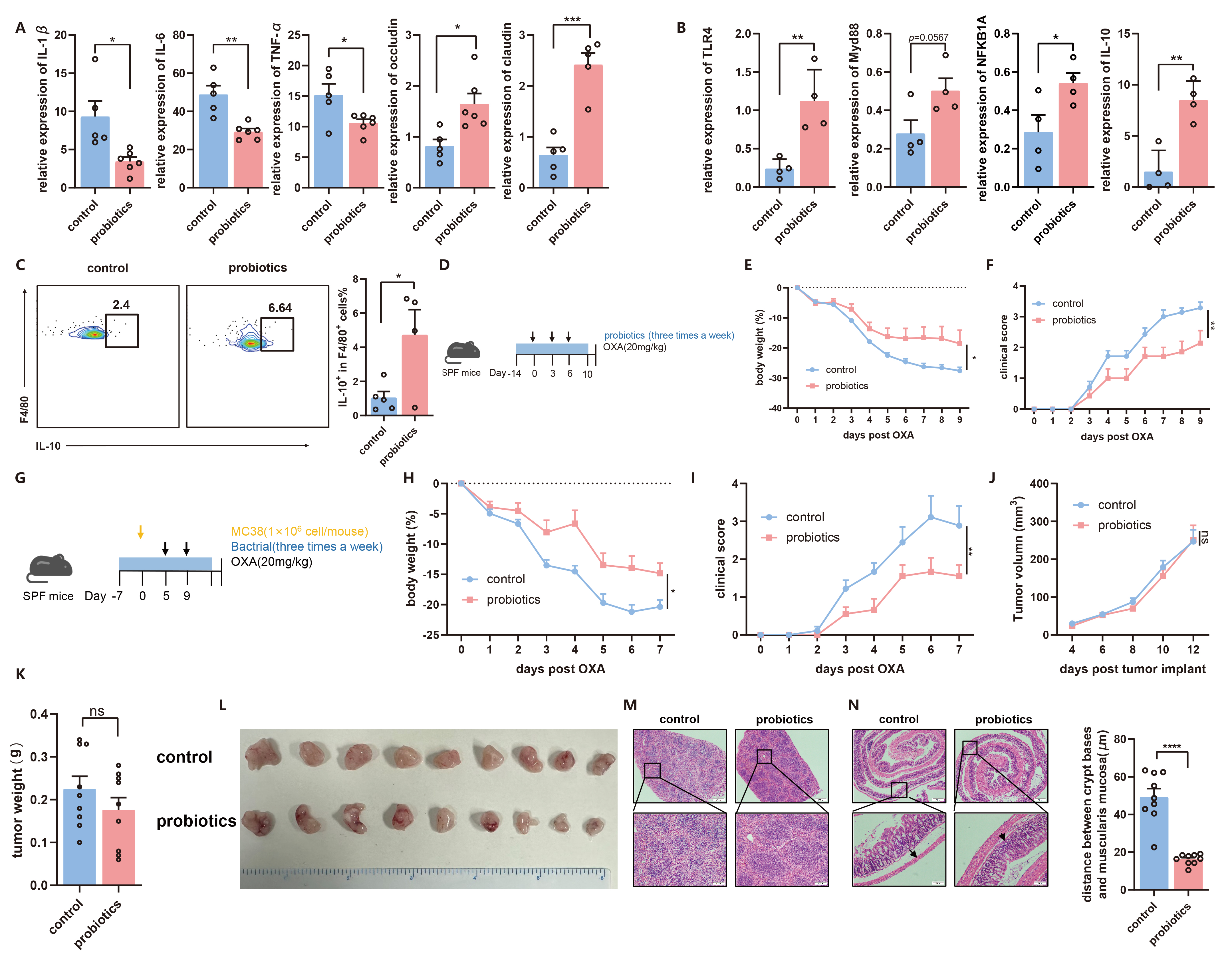

Supplement: Supplemental Material [file KGMI_A_2319511_SM4076.zip › KGMI_A_2319511_supplemental material/figure s4.jpg]

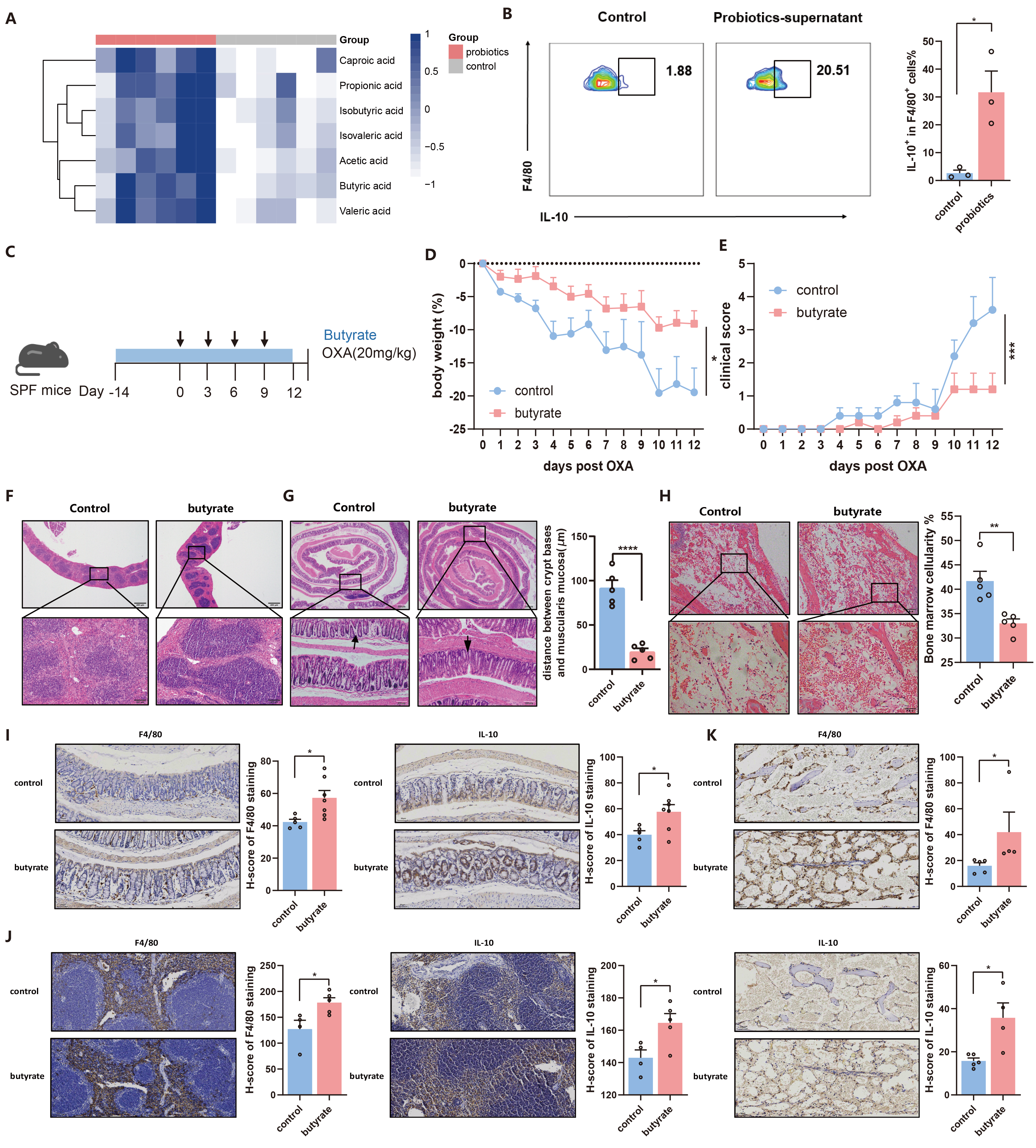

Supplement: Supplemental Material [file KGMI_A_2319511_SM4076.zip › KGMI_A_2319511_supplemental material/figure s5.jpg]

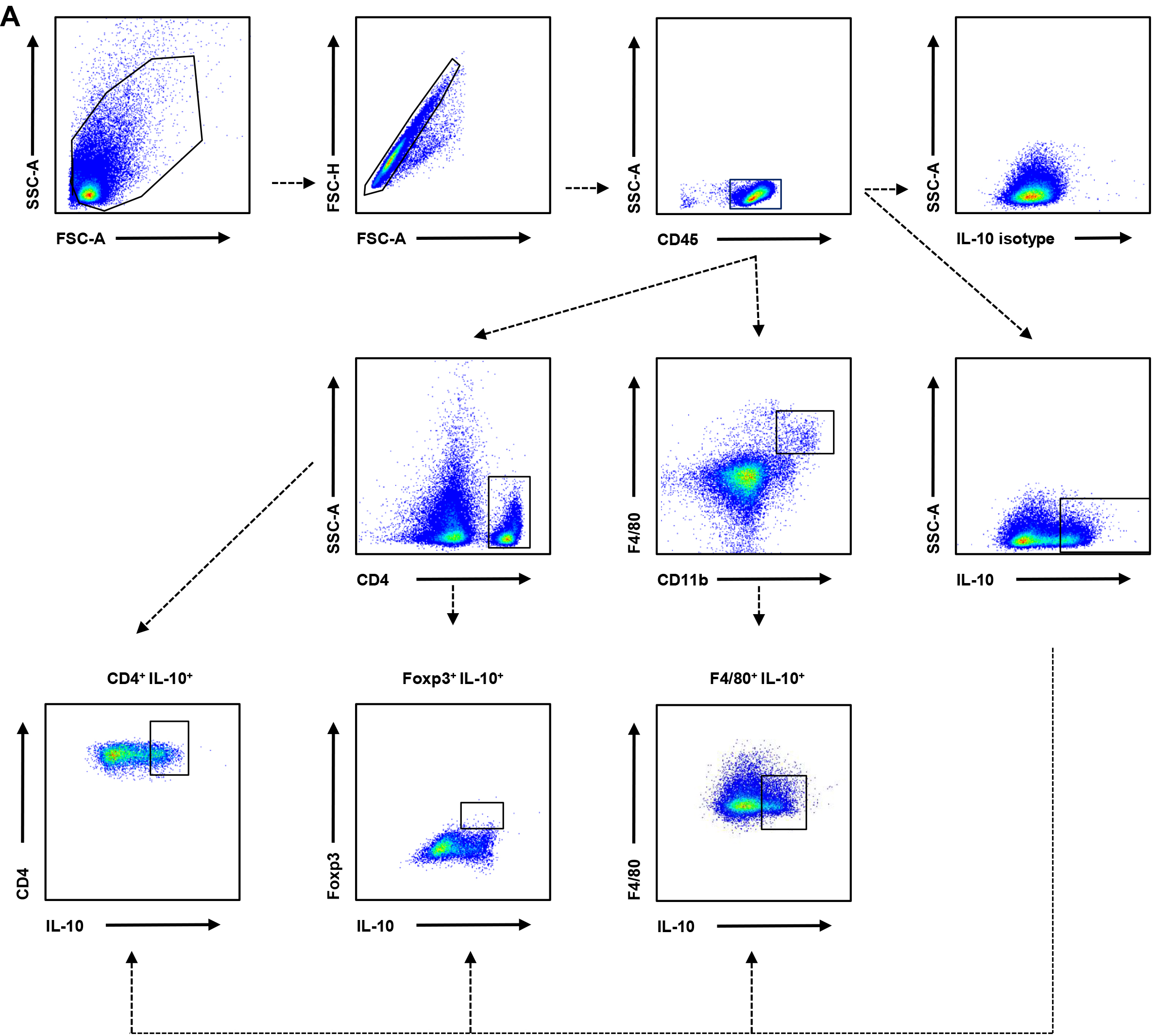

Supplement: Supplemental Material [file KGMI_A_2319511_SM4076.zip › KGMI_A_2319511_supplemental material/figure s6.jpg]
